# Cortistatin as a modulator of inflammatory and mitochondrial dysfunction in Huntington´s disease

**DOI:** 10.1186/s12974-026-03947-4

**Published:** 2026-07-07

**Authors:** Pilar González-García, Ignacio Serrano-Martínez, Eliana Barriocanal-Casado, Alejandro Cuenca-Martagón, Pablo Vargas-Rodríguez, Norma Adán, Irene Forte-Lago, Juan Carlos Morales, Patricia Gomez-Suaga, Júlia Bonet-Freixinet, Silvia Ginés, Luis C. López, Elena González-Rey

**Affiliations:** 1 Institute of Parasitology and Biomedicine Lopez-Neyra (IPBLN), CSIC, Granada, Spain; 2 Department of Physiology, Faculty of Medicine, University of Granada, Granada, Spain; 3 Biomedical Research Center, Institute of Biotechnology, University of Granada, Granada, Spain; 4 Department of Biochemistry, Molecular Biology, and Genetics, Faculty of Nursing and Occupational Therapy, University of Extremadura, Cáceres, Spain; 5 Departament de Biomedicina, Facultat de Medicina i Ciències de la Salut, Institut de Neurociències, Universitat de Barcelona, Barcelona, Spain; 6 Institut d’Investigacions Biomèdiques August Pi i Sunyer (IDIBAPS), Barcelona, Spain; 7 Networked Biomedical Research Center in Neurodegenerative Diseases - Carlos III Health Institute (CIBER-CIBERNED-ISCIII), Madrid, Spain

**Keywords:** Cortistatin, Neuroinflammation, Huntington’s disease, Huntingtin, Mitochondria, Oxidative stress, Neurodegenerative diseases.

## Abstract

**Background:**

Huntington’s disease (HD) is an inherited, fatal neurodegenerative disorder caused by expanded CAG repeats in the Huntingtin gene, leading to progressive motor, cognitive and psychiatric impairment. Despite its monogenic origin, HD pathogenesis is multifactorial, with convergent contributions from mitochondrial dysfunction, oxidative stress, synaptic failure, and chronic neuroinflammation, which drive neuronal vulnerability and degeneration, particularly within the striatum. Current clinical management remains exclusively symptomatic and fails to halt disease progression, highlighting a critical unmet need for strategies targeting fundamental pathogenic mechanisms. Cortistatin, a neuropeptide expressed in the nervous and immune systems, exhibits potent immunomodulatory properties and has recently been implicated in the regulation of mitochondrial function. Notably, cortistatin deficiency is associated with exacerbated systemic and central inflammation, suggesting that impaired cortistatin signaling may contribute to neurodegeneration. However, its role in HD pathophysiology remains unexplored.

**Methods:**

We performed a comprehensive reanalysis of publicly available transcriptomic datasets from HD patients to assess cortistatin expression, followed by validation in experimental HD models. Wild-type and cortistatin-deficient mice treated with 3-nitropropionic acid served as pharmacological HD models, enabling evaluation of cortistatin-dependent disease severity. Behavioral assessments, glial and oxidative markers, and immune factors were evaluated to determine neurological dysfunction and inflammatory responses. Complementary in vitro studies were conducted in striatal neurons expressing mutant huntingtin to examine mitochondrial integrity, inflammatory signaling, metabolic function, and mitochondria-endoplasmic reticulum interactions.

**Results:**

Cortistatin expression was significantly reduced in postmortem HD human brains and across experimental HD models. Cortistatin deficiency exacerbated motor deficits, neuropathological alterations, inflammatory activation, and neuronal vulnerability in HD context. At the cellular level, reduced cortistatin expression was accompanied by amplified inflammatory signaling, disrupted mitochondrial integrity, impaired mitochondria-endoplasmic reticulum interactions, and increased oxidative stress. Conversely, exogenous cortistatin administration attenuated inflammatory mediator production, preserved mitochondrial structure, and improved redox balance in mutant huntingtin-expressing striatal neurons.

**Conclusions:**

Our findings identify cortistatin deficiency as a previously unrecognized contributor to HD pathogenesis and establish cortistatin as a key modulator of neuroinflammation and mitochondrial homeostasis. These results support cortistatin-based strategies as a promising disease-modifying therapeutic avenue for HD and related neurodegenerative disorders characterized by inflammatory activation and mitochondrial impairment.

**Graphical Abstract:**

**Figure:**
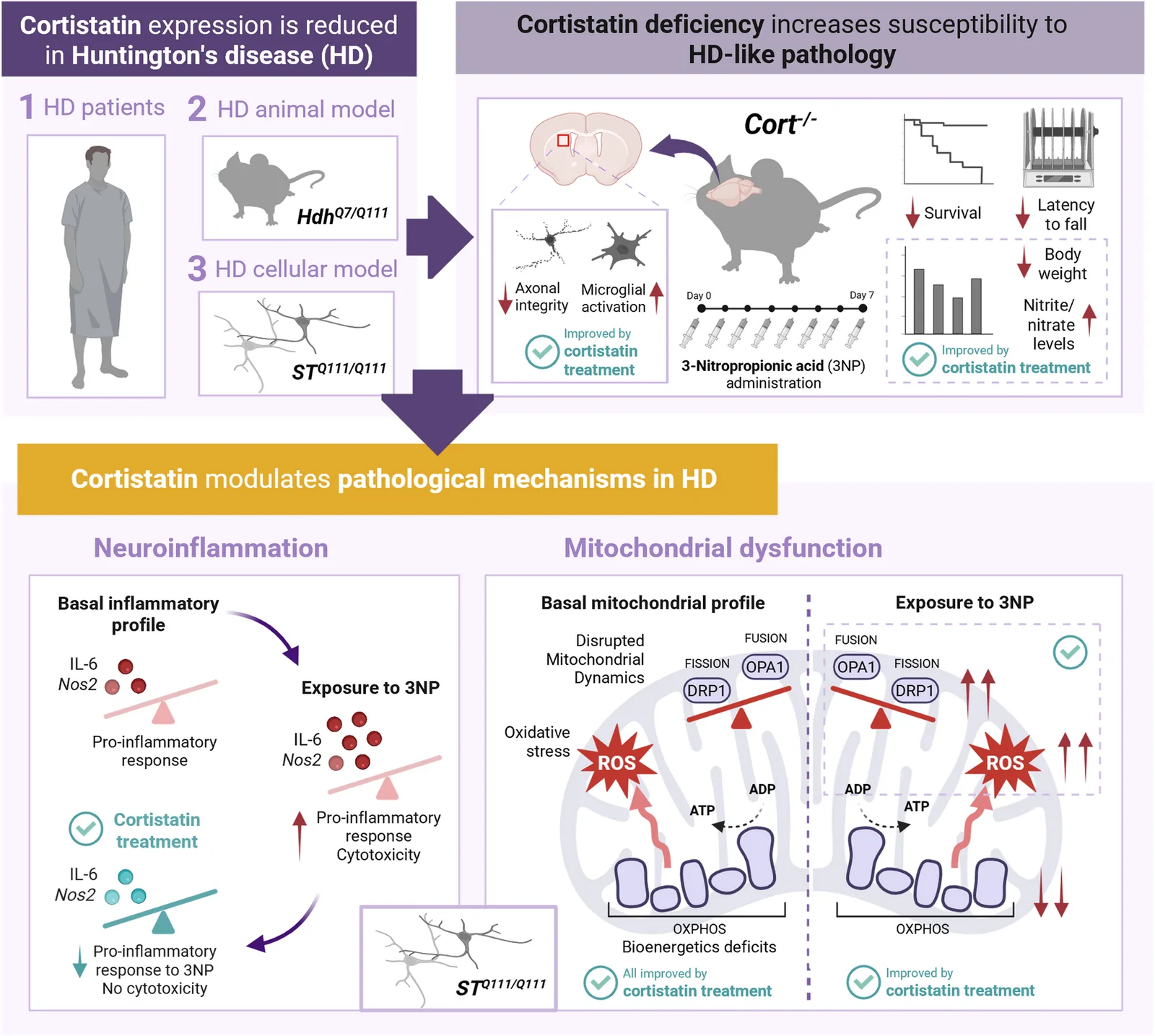

**Supplementary Information:**

The online version contains supplementary material available at https://doi.org/10.1186/s12974-026-03947-4.

## Introduction

Huntington’s disease (HD) is a progressive autosomal dominant neurodegenerative disorder caused by an abnormal expansion of a CAG trinucleotide repeat in exon 1 of the Huntingtin (*HTT*) gene. As a result, a mutant huntingtin protein (mHTT) containing an elongated polyglutamine tract is produced [1, 2]. While wild-type huntingtin is a large cytoplasmic scaffold protein that supports intracellular trafficking, vesicle transport and synaptic function, and is essential for embryonic development and neuronal survival, mHTT misfolds and aggregates, disrupting axonal transport, transcriptional regulation and protein quality-control systems [3, 4]. Normal HTT exerts anti-apoptotic and neuroprotective effects by supporting mitochondrial function, regulating transcription of factors such as BDNF, and promoting efficient autophagy and proteostasis [3–5]. However, mHTT interferes with mitochondrial energy metabolism and calcium handling, thereby increasing oxidative stress [5, 6]. In contrast to the homeostatic role of wild-type HTT in maintaining neuronal resilience, mHTT leads to selective neuronal vulnerability, particularly in the striatum and cerebral cortex, by combining metabolic stress with widespread disturbance of intracellular signaling and giving rise to the characteristic triad of motor, cognitive, and psychiatric disturbances that define the clinical phenotype of HD [7, 8]. In addition, sequestration of wild-type HTT and dominant-negative effects of mHTT produce a partial loss of normal neuroprotective HTT functions, which synergizes with the toxic gain of function to amplify neuronal dysfunction and cell death [9]. Despite advances in understanding the genetic and molecular mechanisms of the disease, currently available therapies remain largely symptomatic, and fail to modify the underlying pathological mechanisms driving neurodegeneration [10].

Among the multifactorial and interconnected processes contributing to HD, mitochondrial dysfunction and neuroinflammation have emerged as central hallmarks of disease progression. Mutant huntingtin disrupts mitochondrial dynamics, compromises electron transport chain activity, alters calcium homeostasis, and promotes oxidative stress, thereby resulting in impaired neuronal energy bioenergetics and survival [11–18]. Concomitantly, neuroinflammation in HD is characterized by the activation of microglia and astrocytes, as evidenced in postmortem human brain tissue [19–21], and by elevated levels of pro-inflammatory cytokines such as interleukin-1β (IL-1β), tumor necrosis factor-α (TNF-α), and interleukin-6 (IL-6) in the plasma and cerebrospinal fluid of HD patients [22, 23]. These immune responses appear early in the disease course, correlate with neuronal loss and clinical severity, and likely contribute to disease progression [20, 22]. Consequently, targeting inflammatory and mitochondrial pathways offers a rational strategy for disease-modifying interventions in HD [24–27].

Cortistatin is a cyclic neuropeptide widely expressed in the central nervous system and by immune cells [28–30]. Although structurally related to somatostatin (SST), cortistatin exhibits distinct biological and pharmacological properties. Both peptides bind to somatostatin receptors (SSTR1-5) but cortistatin also interacts with additional targets, including the ghrelin receptor (GHSR), the Mas gene-related receptor X-2 (MRGPRX2), and a still unidentified cortistatin-specific receptor [29, 31, 32]. These specific receptor interactions confer cortistatin unique neuromodulatory and immunoregulatory capabilities that differ from those of SST. In the context of HD, alterations in somatostatin and SST-positive interneurons have been reported in the striatum and cortex of HD patients and experimental models [33, 34], implicating somatostatinergic dysfunction in altered inhibitory neurotransmission and in disrupted cortical–striatal circuitry, rather than a role in regulating neuroinflammatory and mitochondrial cascades central to HD pathology. In contrast, cortistatin exerts broad immunomodulatory and neuroprotective properties, including suppression of pro-inflammatory cytokines and modulation of glial cell function, in various models of neuroinflammatory and neuroimmune disorders [35–40]. Beyond these functions, growing evidence implicates cortistatin in mitochondrial homeostasis [41–45]. Moreover, cortistatin deficiency promotes a heightened pro-inflammatory state both centrally and peripherally [29, 31, 46]. Interestingly, studies in *Sstr1/Sstr5* double knockout mice revealed neurochemical alterations resembling those observed in transgenic R6/2 HD mice, suggesting that the absence of cortistatin signaling may partially contribute to HD-like molecular abnormalities [47]. These observations underscore the functional divergence between cortistatin and SST and highlight the relevance of investigating cortistatin specifically in the context of HD. Given its dual role, cortistatin may influence HD pathophysiology at multiple levels and reveal novel neuroprotective mechanisms not accessible through somatostatin modulation. However, to date, the specific role of cortistatin in HD remains largely unexplored.

The present study examines cortistatin expression in post-mortem brain tissue from HD patients and in experimental models, and evaluates the impact of cortistatin deficiency on HD-like behavioural and neuropathological features in mice. Furthermore, it explores the molecular interplay between cortistatin and mutant huntingtin, with a focus on neuroinflammation and mitochondrial integrity in striatal neurons. Collectively, our findings aim to provide new insight into HD pathogenesis and identify cortistatin as both a potential biomarker and a mechanism-based therapeutic agent for this neurodegenerative disorder.

## Materials and methods

### Reagents

Mouse cortistatin-29 (CST) was purchased from Bachem (Bubendorf, Switzerland) and was dissolved in physiological saline (0.9% NaCl for in vivo studies; 20 mM phosphate buffer pH 7.0 for in vitro assays). 3-Nitropropionic acid (3NP) (N22908, Sigma) was dissolved in distilled water, and the pH was adjusted to 7.4. Resveratrol (RES, purity > 98%) was kindly supplied by Evolva^®^. The RES derivative, JC21 (resveratrol-3-O-(6’-*O*-octanoyl)-*β*-D-glucopyranoside) was first described by Larrosa et al. [48]. Both compounds were dissolved in DMSO. The final DMSO concentration in each well was adjusted to 0.1%.

### Animals and ethic statement

The experiments reported in this study were approved by the Animal Care and Use Board and the Ethical Committee of the Spanish National Research Council (Animal Care Unit Committee IPBLN-CSIC # protocol CEEA NOV/2016.EGR). They were performed in accordance with the guidelines outlined in Directive 2010/63/EU of the European Parliament concerning the protection of animals used for scientific purposes and reported to comply with the ARRIVE v.2.0 guidelines [49].

This study used *Hdh*^*Q7/Q7*^ (wild-type) and mutant *Hdh*^*Q7/Q111*^ (with targeted insertion of 109 CAG repeats that extends the glutamine segment in murine huntingtin to 111 residues) male mice aged 8 and 10 months. *Hdh*^*Q7/Q7*^ and *Hdh*^*Q7/Q111*^ were maintained on a C57BL/6 genetic background. Wild-type males *Hdh*^*Q7/Q7*^ mice were crossed with heterozygous females *Hdh*^*Q7/Q111*^ mice to generate age-matched *Hdh*^*Q7/Q7*^ and *Hdh*^*Q7/Q111*^ littermates.

This study also employed wild-type (*Cort*^*+/+*^) and transgenic mice deficient in the cortistatin gene (*Cort*^*−/−*^), that were a generous gift of Dr. Luis de Lecea (Stanford University, La Jolla, CA, USA). Mice were generated in a C57BL/6 background and backcrossed with C57BL/6 mice for ten generations, as previously described [31]. *Cort*^*+/+*^ and *Cort*^*−/−*^ male mice 3 months old were used in all experiments. Animals were housed in a controlled-temperature/humidity environment (22 ± 1 °C, 60–70% relative humidity) with a 12 h light/dark cycle (lights on at 7:00 a.m.) and were fed with rodent chow (Global Diet 2018, Harlan, KY, USA) and tap water ad libitum. Mice were randomly assigned to different experimental groups (5–8 mice per group and cage).

### Induction of the 3-nitropropionic acid model of Huntington’s disease

The pharmacological model of HD was performed as previously described [50, 51]. Briefly, to assess survival, 3NP was injected intraperitoneally once daily. The first injection was 30 mg/kg, and the dose was increased daily by 15% over the previous concentration. To analyse neuronal damage induction, 3NP was injected intraperitoneally twice a day, with 8–10 h between injections, for 5 consecutive days. The doses administered were: 2 × 20 mg/kg, 2 × 20 mg/kg, 2 × 40 mg/kg, 2 × 40 mg/kg, and 2 × 60 mg/kg (cumulated dose: 360 mg/kg in 5 days). Cortistatin was administered daily at a concentration of 0.3 mg/kg, based on previous data [39, 40]. Naive mice injected with PBS (saline) instead of 3NP or cortistatin were used as controls. Mice were euthanized using CO_2_ inhalation. All treatments were administered in a volume of ≈ 100 µL and always after behavioural evaluations.

### Behavioural test

To evaluate motor function and ataxia, the rotarod test was performed using a rotating cylinder apparatus (Ugo Basile, Gemonio, Italy) with a gradual acceleration protocol. The cylinder accelerated from 4 rpm to 40 rpm and then maintained a constant speed of 40 rpm until reaching a 300 s time limit. To ensure reliable results and exclude motivational factors, such as early falls, a training phase was conducted three days prior to the start of the experiment [52]. Mice were tested daily for 6 days. Body weight was recorded simultaneously each day.

### Tissue collection

At the end of the 3NP intoxication experiment, mice were sacrificed in a CO_2_ chamber and blood and brain tissue were immediately collected. Blood was centrifuged for serum recovering, and collected serum samples were stored at − 20 °C from obtaining until the analysis of cytokine content. Brains were collected and post-fixed in 4% PFA for 24 h at 4 °C, and then cryoprotected in 30% sucrose for 48 h at 4 °C. Subsequently, brains were frozen in isopentane on dry ice at − 25 °C and stored at − 80 °C until further immunodetection. Brains processed to qRT-PCR assay were directly frozen in liquid nitrogen.

### Cell culture

Conditionally immortalized wild-type *STHdh*^*Q7/Q7*^ (*ST*^*Q7/Q7*^) and mutant *STHdh*^*Q111/Q111*^ (*ST*^*Q111/Q111*^) striatal neuronal progenitor cell line were used. These cells, first characterized by Trettel et al. [53], express endogenous levels of normal and mutant Htt (mHtt) with 7 and 111 polyQ, respectively, and were grown and maintained in high glucose Dulbecco’s Modified Eagle Medium (Sigma) containing 10% fetal bovine serum, 1% Penicillin-Streptomycin solution (Gibco), 2 mM L-glutamine (Gibco), 1 mM sodium pyruvate (Gibco), 1% non-essential amino acids (Gibco) and 400 µg/ml G418 (Gibco). Cells were maintained at 33 °C in a humidified atmosphere containing 5% CO_2_ and treated with 100 nM CST, 10 µM RES and 10 µM JC21 and/or 2.5 mM 3NP. Treatments were generally applied for 24 h, although in specific cases 48 h or 7-day treatments were used, as indicated.

### Cell viability assay

Cell viability was assessed using the Cytotoxicity Detection Kit (Roche Applied Science, 04744934001), which quantifies lactate dehydrogenase (LDH) released as a marker of membrane integrity and cytotoxicity. After treatment, 50 µL of cell culture supernatant from each well were transferred to a 96-well plate, mixed with 50 µL of reaction mixture and incubated for 30 min at room temperature in the dark. The reaction was stopped by adding 25 µL of the stop solution and the absorbance was measured at 490 nm (reference wavelength of 630 nm). As a negative control (0% cell death), culture medium incubated under the same conditions but without cells was used. As a positive control (100% cell death), cells were lysed by adding Triton X-100. Cell viability was calculated as the percentage of viable cells relative to total LDH release.

### Immunofluorescence

For brain tissue, collected brains were cryosectioning in 20 μm free-floating sections that were washed with PBS and blocked and permeabilized with PBS containing 1% BSA, 0.1% Triton X-100, and 5% goat serum for 60 min at room temperature (RT). Following this, sections were incubated overnight (O/N) at 4 °C with primary antibodies anti-Iba1 (1:2000, Wako), anti-GFAP (1:5000, Dako), anti-NeuN (1:500, Millipore), or anti-MBP (1:500, Millipore) in PBS containing 1% BSA, 0.1% Triton X-100, and 5% goat serum. After extensive washing with PBS, sections were incubated with the corresponding secondary Alexa Fluor-conjugated antibodies (1:1000, Invitrogen) and DAPI in PBS containing 1% BSA, 0.1% Triton X-100, and 2% goat serum for 60 min. Sections were mounted with Mowiol.

For in vitro assays, after exposure to treatments, cultured neurons were fixed with 4% paraformaldehyde (Sigma) for 10 min at RT. After extensive washing with PBS, cells were incubated with 30 mM glycine (Sigma) for 5 min, washed again, and permeabilized with PBS containing 0.1% Triton X-100 (Aplichem) for 15 min at RT for TOM20 staining and 0.3% Triton X-100 for OPA1 staining. Cells were then blocked with 10% goat serum, 3% BSA, and 0.1% Triton X-100 in PBS for 60 min at RT, and overnight incubated (4 °C) with primary polyclonal antibodies anti-rabbit TOM20 (1:500, Proteintech), and for 48 h with anti-rabbit OPA1 (1:100, Proteintech). Next, cells were washed with PBS followed by 60 min incubation at RT with secondary Alexa Fluor™ 594-conjugated goat anti-rabbit IgG antibody (1:1000, Invitrogen). After repeated washes, nuclei were DAPI-counterstained (1 µg/ml) for 5 min RT, rinsed with PBS, and mounted with Mowiol (Sigma).

Images were captured using a LEICA DMi8 S Platform Life-cell microscope at 20x magnification and a confocal Leica TCS SP8 STED microscope at 63x magnification. Image fluorescence analyses were performed with ImageJ/Fiji free software.

### Morphometric analysis of glial phenotypes

We have performed a comprehensive morphological characterization using an automated and blinded pipeline. To ensure objectivity and eliminate observer bias, image processing was conducted using ImageJ/Fiji software following a systematic, macro-based protocol adapted from established studies [39, 54, 55]. All images were converted to 8-bit grayscale and subjected to automated noise reduction and contrast enhancement, while binarization was performed using a fixed, pre-determined threshold applied consistently across all experimental groups. Manual refinement was strictly limited to the removal of non-cellular artefacts and debris to ensure the accuracy of the segmentation. Once processed, morphometric parameters were extracted automatically using the *Analyze particles*,* Skeletonize* and *Fractal Analysis* plugins [56], allowing for the objective quantification of total area, perimeter, number and length of primary and terminal branches, fractal dimension, circularity and span ratio.

### Determination of inflammatory factors

The concentration of inflammatory mediators and cortistatin was determined by sandwich ELISA assay. Briefly, maxisorb plates (Millipore) were coated overnight at 4 °C with the capture anti-IL-6 (BD Pharmigen) antibodies. Next day, plates were washed with PBS/0.05%-Tween20, blocked with PBS/10% FBS for 2 h, and incubated at 4 °C overnight with the samples and recombinant standards. Biotinylated detection antibodies (Serotec) were added for 1 h at RT. After washing, plates were incubated with avidin-HRP (Sigma) (1:500 in blocking buffer) for 1 h and developed with ABTS substrate (Sigma). Absorbance was measured at 405 nm with a spectrophotometer (Molecular Devices) and concentrations were determined according to the standard curve generated at the same time. For nitrite quantification, equal volumes of culture supernatants and Griess reagents (0.1% N-[naphthyl]ethylenediamine dihydrochloride in water and 1% sulphanilamide in 5% H_3_PO_4_) were loaded into a 96-well plate. Absorbance was measured at 540 nm in a spectrophotometer, and the amount of nitrite was calculated from a NaNO_2_ standard curve.

Contents of cortistatin-29 was assayed using a specific competitive ELISA for cortistatin (Phoenix Pharmaceuticals, Karlsruhe, Germany) following the manufacturer´s instructions.

### Gene expression analyses

Total RNA from tissue and cultured cells was extracted using the TriPure reagent (Roche). Then, cDNA synthesis was conducted on 20 ng RNA per sample using the RevertAid First Strand cDNA Synthesis Kit (ThermoFisher) with random hexamers in a CFX Connect QPCR System (Biorad) under the following conditions: incubation at 25 °C/5 min, reverse transcription at 42 °C/60 min, inactivation at 70 °C/5 min. Gene expression analysis was conducted using conventional qPCR. SensiFast Sybr No-Rox kit 1x (Bioscience) was added to the cDNA, along with the corresponding reverse and forward primers for each gene, and nuclease-free water to a final volume of 20 µl. The CFX96 thermocycler (BioRad) was used to conduct the reaction under the following conditions: 94 °C/5 min (polymerase activation) followed by 40 cycles at 94 °C/30 s (denaturation), 58 °C/60°C/62°C/30 s (primer annealing temperature depending on the gene), and 72 °C/30 s (extension). The comparative threshold cycle (Ct) method was performed for quantification. Each gene Ct values were normalized to those of the housekeeping gene *Gapdh* within each PCR reaction. The specific primers for each gene are provided in Table S1 (Supplementary File 2).

### Western blot analyses

For western blot analyses, a glass-Teflon homogenizer was used to homogenize mouse brain samples at 1100 rpm in T-PER^®^ buffer (Thermo Scientific) with protease and phosphatase inhibitor cocktail (Thermo Scientific). Homogenates were sonicated and centrifuged at 1000 g for 5 min at 4 °C, and the resultant supernatants were used for the analyses. For western blot analyses in cells, cell pellets were re-suspended in RIPA buffer with protease and phosphatase inhibitor cocktail (Thermo Scientific) and centrifuged at 10,000 rpm for 10 min at 4 °C. About 40 µg of protein from the tissue sample extracts and 20 µg of protein from cell samples were electrophoresed in 12% Mini-PROTEAN TGX™ precast gels (Bio-Rad) using the electrophoresis system mini-PROTEAN Tetra Cell (Bio-Rad). Proteins were transferred onto PVDF 0.45-µm membranes using a Trans-blot Cell (Bio-Rad) and probed with target antibodies. Fluorescence signal was detected using the Odyssey CLx Near-Infrared Fluorescence Imaging System (LI-COR Biosciences, Lincoln, NE, USA). Protein band intensity was quantified using ImageJ software (NH, Bethesda, USA) and normalized to the corresponding loading control band present on the same membrane, VDAC or TOM20 for mitochondrial proteins and β-ACTIN for cytosolic proteins. To allow comparison across independent blotting experiments, normalized values were expressed relative to wild-type mice or control cells. Original western blot membranes are described in Supplementary File 1.

The following primary antibodies were used: anti-MBP (Abcam, ab218011), anti-SOD1 (Proteintech, 10269-1-AP), anti-SOD2 (Proteintech, 24127-1-AP), anti-CAT (Proteintech, 21260-1-AP), anti- OPA1 (Proteintech, 27733-1‐AP), anti-DRP1 (Proteintech, 12957-1-AP), anti-VDAC (Abcam, ab14734), anti-TOM20 (Proteintech, 11802‐1‐AP) and anti-ACTIN (Proteintech, 66009-1-Ig).

### cAMP assay

Intracellular cAMP levels in striatal neurons were measured using the Parameter™ cAMP Assay Kit (R&D Systems, Cat. # KGE002B) in the presence and absence of cortistatin and 3NP for 1 h, or forskolin (10 µM) for 15 min (as positive control for cAMP induction), in the presence of phosphodiesterase inhibitor 3-isobutyl-1-methylxanthine (1 µM). After the treatments, cells were washed in cold PBS, resuspended in Cell Lysis Buffer 5 (1:5) at 1 × 10⁷ cells/mL, and lysed by two freeze-thaw cycles at ≤ -20 °C. Lysates were clarified by centrifugation at 600 × g for 10 min at 2–8 °C. Standards (3.75–240 pmol/mL) and samples were assayed in duplicate. Following sequential incubations with primary antibody (1 h) and cAMP conjugate with samples (2 h) at room temperature with orbital shaking, TMB substrate was added for 30 min and the reaction was stopped with 2 N sulfuric acid. Absorbance was read at 450 nm.

### Mitochondrial characterization

#### Mitochondrial DNA quantification

Mitochondrial DNA was quantified by real-time PCR in a Stratagene Mx3005P Real-Time PCR System (Agilent Technologies Inc., CA, USA). We used primers and probe complementary to sequences of the nuclear gene 18S (TaqMan Gene Expression Assays Hs99999901_s1, Applied Biosystems) and the sequences of the 12S gene for mtDNA: (5’CCACGGAAACAGCAGTGATT3’) for the forward and (5’CTATTGACTTGGGTTAATCGTGTGA3’) for reverse primers, and 50nM mtDNA probe (12 S RNA Oligo 5’TGCCAGCCACCGCG3’). Results are expressed as a ratio of mtDNA/nDNA copy.

#### Mitochondrial bioenergetics

Oxygen consumption rate (OCR) and extracellular acidification rate (ECAR) were measured in neurons with a XF24 Extracellular Flux Analyzer (Seahorse Bioscience, Billerica, MA, USA). Each cell line was seeded in the wells of a XF 24-well cell culture microplate (Seahorse Bioscience) at a density of 6 × 10^4^ cells/well in 250 µL of DMEM and incubated for 16 h at 37 °C in 5% CO_2_ atmosphere. After replacing the growth medium with 525 µL of XF DMEM pH 7.4 (Agilent) pre-warmed at 37 °C, cells were preincubated for 1 h in a CO_2_ free incubator before starting the assay procedure [57]. For OCR measurement, assay medium was supplemented with glucose, glutamine and sodium pyruvate. After baseline measurements, OCR was analyzed after sequentially adding to each well 75 µL of oligomycin, 75 µl of carbonyl cyanide 4-(trifluoromethoxy) phenylhy-drazone (FCCP) and 75 µl of rotenone and antimycin, to reach working concentrations of 1 µM, 0,75 µM, and 1 µM, respectively [57]. All data were expressed in pmol/min/1000 cells and represented as relative to the mean of the three measurements before oligomycin injection in *ST*^*Q7/Q7*^ CTR experimental group. For ECAR measurement, assay medium was supplemented with glutamine. The assay was conducted by sequential injection of the following compounds into each well: 75 µL of glucose, 75 µl of oligomycin and 75 µl of 2-deoxy-D-glucose (2-DG), to reach working concentrations of 10 mM, 1 µM, and 50 mM, respectively. All data were expressed in mpH/min/1000 cells and represented as relative to the mean of the three measurements before glucose injection in *ST*^*Q7/Q7*^ CTR experimental group.

#### Quantification of CoQ_9_ and CoQ_10_ levels

Following lipid extraction from cultured cells [58], CoQ_9_ and CoQ_10_ levels were quantified using reversed-phase high-performance liquid chromatography (HPLC) coupled to electrochemical detection, as previously detail [58]. For quantification, a calibration curve generated with CoQ_9_ and CoQ_10_ standards was used to estimate concentrations. Data were normalized to total protein content and expressed as ng CoQ/mg protein.

#### Determination of respiratory complex activities

Cell pellets (of ~ 4 × 10^6^ cells) were thawed, suspended in 350 µL of buffer (250 mM sucrose, 2 mM EDTA 100 mM Tris-HCl, pH 7.4) and immediately sonicated to disrupt all cellular membranes, including the mitochondrial membrane. Protein concentration was determined by Bradford using BSA as standard. The activity of complex II (succinate dehydrogenase, expressed as µmoL of reduced DCIP/min/mg protein) was measured by following reduction of 2,6-dichlorophenol-indophenol (DCIP) at 600 nm for 1 min at 30 °C. Complex IV activity (cytochrome c oxidase, in µmoL of oxidized cytochrome c/min/mg protein) was measured as the disappearance of reduced cytochrome c at 550 nm [59].

#### Measurement of ROS production

ROS production was measured using 100 µM of 2′,7′-dichlorofluorescin diacetate (DCFH-DA) probe (Sigma-Aldrich, Madrid, Spain), according to the previously described method [60]. Inside the cell, DCFH-DA is oxidized by ROS and is transformed to fluorescent 2’,7’-dichlorofluorescein (DCF). Fluorescence was then measured with the microplate fluorescence reader (Bio-Tek Instruments, Inc., Winooski, VT, USA) for 45 min every 5 min, at 485 nm to excitation and 530 nm to emission.

#### Mitochondrial morphology assessment

Briefly, mitochondrial architecture was visualized by TOM20 immunostaining (explained in immunofluorescence section) using a confocal Leica TCS SP8 STED microscope at 63× magnification. Mitochondrial morphology was classified into four categories based on established structural criteria [61, 62]: (i) Tubular: elongated, interconnected mitochondria forming a filamentous network, without visible discontinuities; (ii) Fragmented: predominantly punctate, short, rounded, mitochondria that are physically disconnected from one another and distributed as across the cytoplasm; (iii) Mixed: a combination of elongated and shorter mitochondria within the same cell, representing and intermediate dynamic state; (iv) Swollen: enlarged, spherical mitochondrial with visible increased diameter suggesting matrix swelling and membrane integrity compromise. For quantification, each cell in the field was individually assigned to one of the four morphological categories based on the predominant mitochondrial structural phenotype observed. The proportion of cells with mitochondria belonging to each morphological category was then calculated per field and per experimental group. Classification was performed in 4–5 regions of interest (ROIs) per field, 2 different fields per culture, across 3 independent experiments.

#### Analysis of mitochondria-ER contacts by Proximity Ligation Assay (PLA)

Proximity ligation assay (PLA) was performed using Duolink^®^ detection kit with green fluorophore λ excitation 495 nm/λ emission 527 nm. After fixation and blocking, cells were first incubated with primary antibodies IP3R3 (1:500, Millipore) and VDAC1 (1:250, Millipore) overnight. After washing with PBS, PLA probes anti-mouse PLUS and anti-rabbit MINUS were added for 1 h and hybridization was amplified by rolling circle amplification. Finally, cells were incubated overnight with rhodamine phalloidin-conjugated antibody (1:250, Invitrogen) to stain actin cytoskeleton in striatal neurons and then nuclei were stained with 4 ‘6-diamidino-2-fenilindol (DAPI). Digital images were acquired using a 63x objective and stacks of 0.3 µm. Fluorescent dots were analyzed using the plugin “Analyze particles” of ImageJ. Number of particles were normalized per cell. As a negative control, one of the primary antibodies was omitted.

### Integration of external human datasets for the analysis of cortistatin-related gene expression

Cortistatin-related gene expression levels were obtained from the Gene Expression Omnibus database (GEO), incorporating datasets from human samples with HD. We analysed data from peripheral blood (GSE51799) [63] and post-mortem brain tissue (GSE242195) [64] from healthy controls and HD patients. For the peripheral blood dataset, the analysis was performed on total RNA extracted from blood samples of 91 mutation carriers (including 27 presymptomatic and 64 symptomatic individuals) and 33 control subjects. In the post-mortem brain tissue dataset, dissection was performed on regions with varying degrees of neuropathological involvement, including the severely affected caudate nucleus, and the relatively less affected cingulate cortex and nucleus accumbens. 76 samples derived from 10 control individuals and 20 HD patients were used in this study. Cortistatin-related gene expression levels were reported as normalized RPKM.

### Statistical analysis

Data are presented as the mean ± SEM. Statistical differences between two groups were conducted using the unpaired Student’s t test for normal distribution and parametric data. For comparing three or more groups that displayed normal distribution and parametric data, one-way ANOVA with Fisher´s LSD post-hoc test for multiple comparisons was conducted. Differences in survival distributions between groups were assessed with the log-rank (Mantel–Cox) test. All statistical analyses were performed using GraphPad Prism v10 software, with P-values < 0.05 (one-tailed) considered statistically significant.

## Results

### Cortistatin-related gene networks are dysregulated in Huntington’s disease

**Fig. 1:**
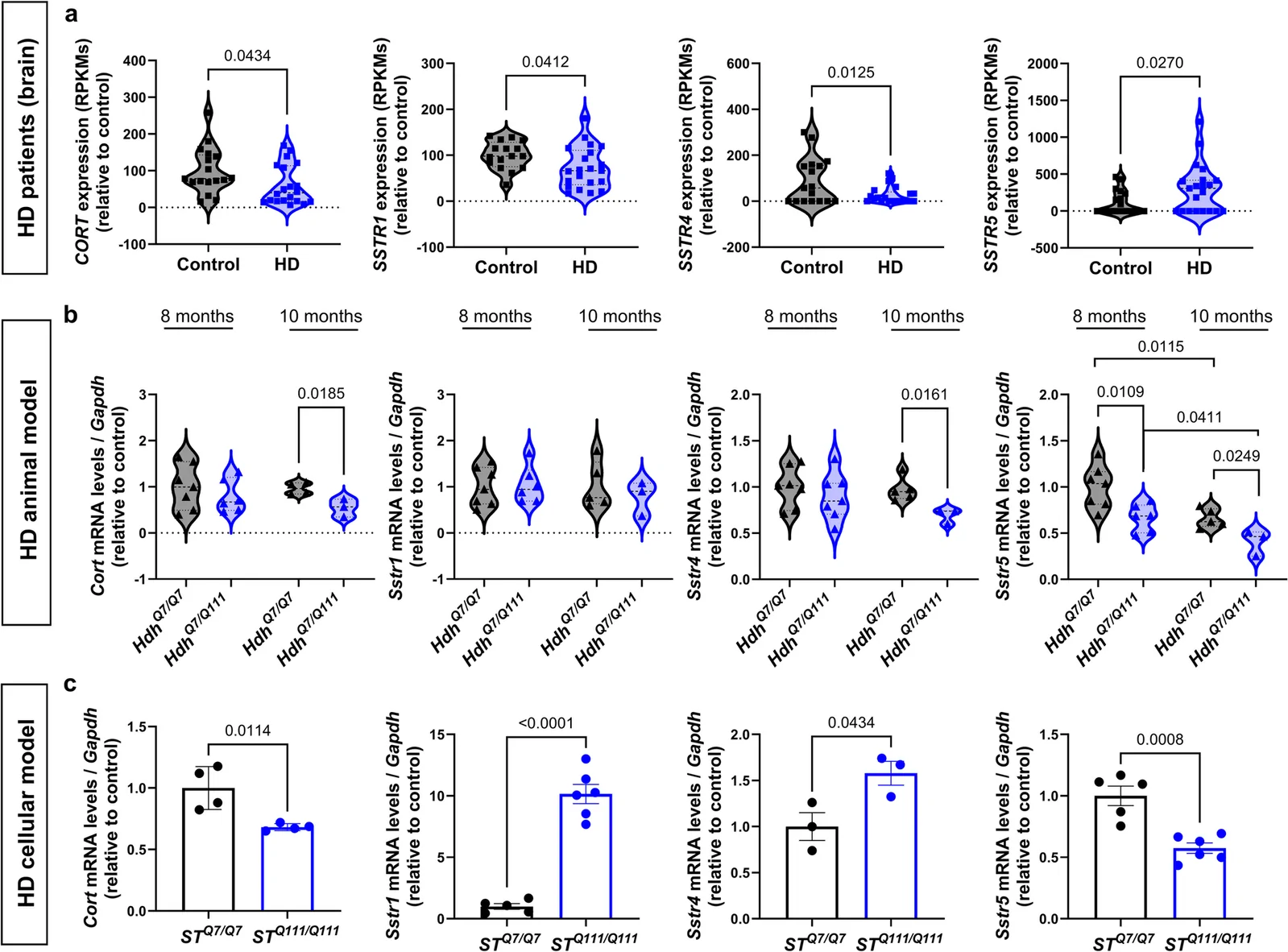
Dysregulation of cortistatin-related genes in Huntington´s disease across human tissue and experimental models. Cortistatin-related gene expression was evaluated in Huntington’s disease (HD) human postmortem tissue, in HD mouse model, and in striatal neurons. **a** Gene expression of *CORT*, *SSTR1*, *SSTR4* and *SSTR5* in postmortem brain samples from HD patients (*N* = 20) and healthy controls (*N* = 17) quantified as normalized RPKM values. The data were obtained from the GEO dataset GSE242195. For region-specific data, see Fig. S1 (Supplementary File 2), which presents cortistatin-related gene expression stratified by brain region: caudate nucleus (Fig. S1a), cingulate cortex (Fig. S1b), and nucleus accumbens (Fig. S1c). **b** Gene expression levels of *Cort*, *Sstr1*, *Sstr4* and *Sstr5* in the striatal tissue of *Hdh*^*Q7/Q7*^ and *Hdh*^*Q7/Q111*^ mice at 8 months (*N* = 7) and 10 months of age (*N* = 3-5), quantified by real-time qPCR and normalized to *Gapdh* expression. **c** Gene expression levels of *Cort*, *Sstr1*, *Sstr4* and *Sstr5* in *ST*^*Q7/Q7*^ and *ST*^*Q111/Q111*^ striatal neurons quantified by real-time qPCR and normalized to Gapdh expression (*N* = 3-6 cell cultures). Data represent the mean ± SEM. Statistical analyses were performed using two-tailed unpaired Student’s *t*-test. *CORT/Cort*: cortistatin, HD: Huntington’s disease, *SSTR1/Sstr1*: somatostatin receptor 1, *SSTR4/Sstr4*: somatostatin receptor 4, *SSTR5/Sstr5*: somatostatin receptor 5

First, we examined the expression profiles of cortistatin-related genes in both human patients and murine HD models. Cortistatin gene expression values from human samples were retrieved from publicly available GEO datasets, as described in the Methods section. Our analysis revealed that post-mortem brain tissue from HD patients exhibited a significant reduction in cortistatin expression compared with healthy controls (Fig. 1a).

Region-specific analysis demonstrated that the caudate nucleus, one of the most severely affected structures in HD and the major component of the striatum, displayed the largest decrease in cortistatin expression (Fig. S1a, Supplementary File 2). This reduction was not observed in the cingulate cortex and nucleus accumbens, regions typically subject to milder neuropathological involvement (Fig. S1b, c, Supplementary File 2). In contrast, cortistatin expression in peripheral blood did not differ between HD patients and control subjects (Fig. S2a, Supplementary File 2). Similarly, genes encoding structurally or functionally related neuropeptides, such as somatostatin (SST) and ghrelin (GHRL), showed no significant changes (Fig. S2b, Supplementary File 2). We next examined the transcriptional profiles of cortistatin receptors. HD brain tissue displayed decreased expression of SSTR1 and SSTR4, accompanied by increased expression of SSTR5 (Fig. 1a). This pattern was consistently observed across individual brain regions (Fig S1, Supplementary File 2), whereas expression of additional cortistatin-related receptors (SSTR2, SSTR3, and GHSR) remain unchanged (Fig. S2b, Supplementary File 2).

To investigate whether the alterations observed in human samples are recapitulated in a preclinical model, we assessed cortistatin-related gene expression in the *Hdh*^*Q7/Q111*^ knock-in mouse model of HD, which expresses mutant huntingtin containing 111 CAG repeats [65–70]. Striatal tissue from 10-month-old *Hdh*^*Q7/Q111*^ mice, an age at which motor and cognitive impairments are established, exhibited significantly reduced cortistatin transcript levels, whereas no changes were observed at earlier ages (Fig. 1b). ELISA quantification confirmed a significant decrease in cortistatin protein levels in the striatum of mutant mice only at 10 months (Fig. S2c, Supplementary File 2). A similar age-dependent reduction was detected in the hippocampus, while cortistatin levels in the cortex remained unchanged. Notably, hippocampus displayed the highest cortistatin content in both genotypes at all ages examined (Fig. S2c, Supplementary File 2). Consistent with findings in human tissues, *Hdh*^*Q7/Q111*^ mice showed dysregulated expression of cortistatin receptors *Sstr4 and Sstr5*, particularly evident at 10 months (Fig. 1b). Parallel analyses in striatal neurons expressing mutant huntingtin (*ST*^*Q111/Q111*^) further confirmed altered expression of cortistatin and its receptors (Fig. 1c). Cortistatin protein levels were also significantly reduced in *ST*^*Q111/Q111*^ compared with control neurons, with a similar decrease observed in wild-type cells after 3NP treatment (Fig. S2d, Supplementary File 2), corroborating the gene expression data and providing protein-level validation of cortistatin dysregulation both in in vitro and in vivo HD models. Functionally, *ST*^*Q111/Q111*^ cells displayed increased susceptibility to cytotoxicity induced by 3-nitropropionic acid (3NP), which was associated with reduced cortistatin availability and receptor imbalance (Fig. S2d, Supplementary File 2). Collectively, these findings suggest that the pathological context of HD is characterized by a consistent decrease in cortistatin expression accompanied by dysregulation of its receptor network.

### Cortistatin deficiency increases susceptibility to Huntington´s disease-like pathology

**Fig. 2:**
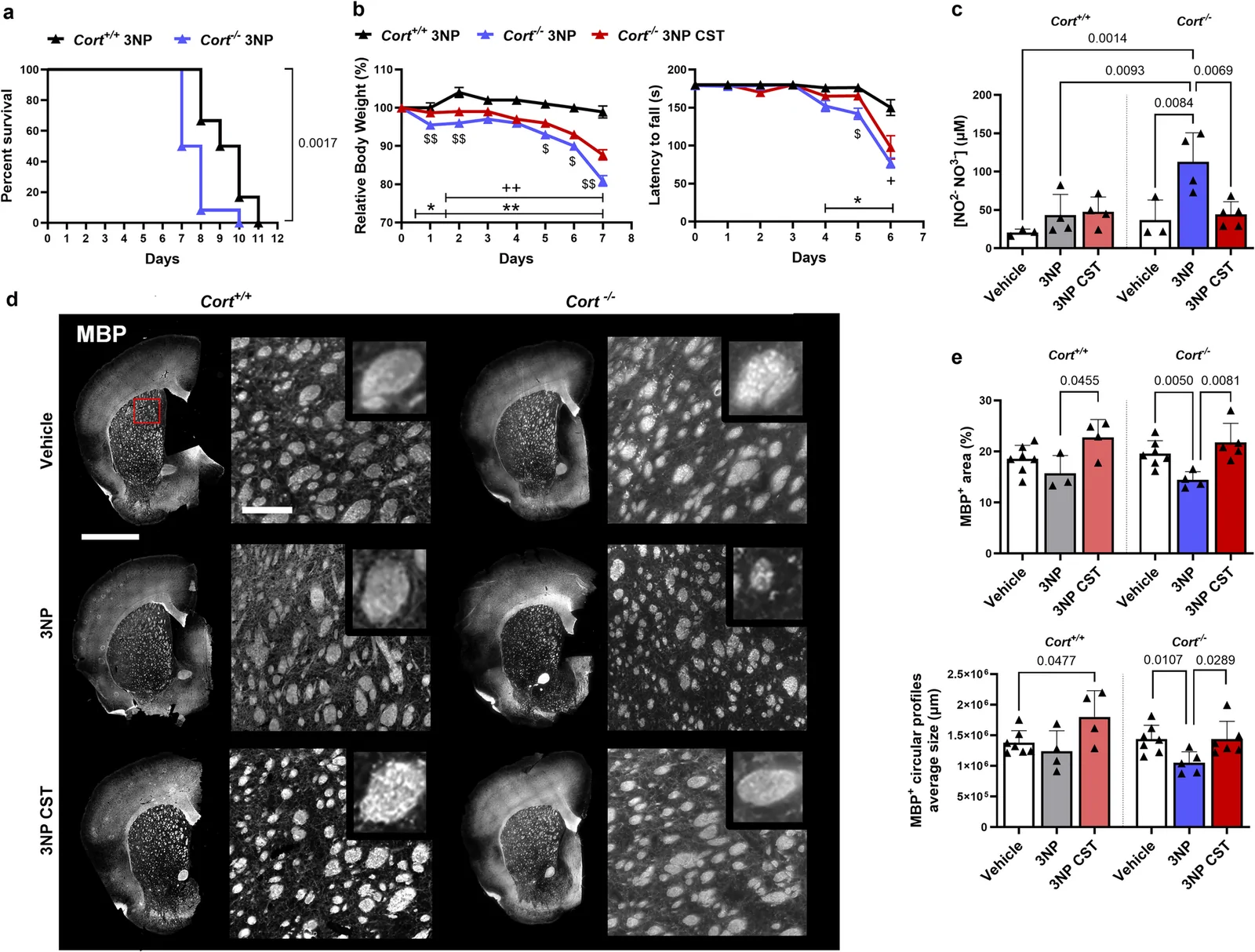
Lack of cortistatin exacerbates 3NP-induced HD-like phenotype in mice. **a** Survival curves of *Cort*^*+/+*^ (*N* = 12) and *Cort*^*-/-*^ (*N* = 24) mice following daily intraperitoneal administration of 3NP, starting at 30 mg/kg with a 15% daily dose escalation. **b**-**e** To evaluate clinical signs and pathological mechanisms, 3NP was intraperitoneally injected to *Cort*^*+/+*^ and *Cort*^*-/-*^ mice twice a day for 5 consecutive days (cumulated dose: 360 mg/kg; see methods). Cortistatin (CST) (0.3 mg/kg) was daily administered. **b** Relative body weight (left) and motor coordination assessed by rotarod performance (right) were recorded (*N* = 4-5 mice); **c** serum nitrite levels were quantified by the Griess assay (*N* = 3-5 mice); **d** representative coronal brain sections were immunostained for MBP (white) to identify myelin. Scale bar: 1500 µm (whole section) and 50 µm (higher magnification); **e** quantification in the striatum of MBP-positive area (top, red squares) and mean size of MBP^+^ circular profiles (bottom, reflecting myelin structural integrity) (*N* = 3-7 mice). Data are shown as mean ± SEM. Differences in survival distributions between groups in panel (**a**) were assessed with the log-rank (Mantel–Cox) test. Statistical analyses were performed using two-tailed unpaired t-test in panel (**b**); detailed analyses are provided in Tables S2 and S3. One-way ANOVA with Fisher´s LSD post-hoc test in panels (**c**, **e**). ^*/+/$^*p* < 0.05; ^**/++/$$^*p* < 0.01; *Cort^*+/+*^ 3NP vs *Cort*^*-/-*^ 3NP, ^*+*^*Cort*^*+/+*^ 3NP vs *Cort*^*-/-*^ 3NP CST, ^$^*Cort*^*-/-*^ 3NP vs *Cort*^*-/-*^ 3NP CST. CST: cortistatin, MBP: myelin basic protein, NO₂⁻/NO₃⁻, nitrite/nitrate, 3NP: 3-nitropropionic acid

To investigate the functional contribution of cortistatin to HD-related pathology, we employed the 3NP mouse model, a widely validated pharmacological model that reproduces key neuropathological and metabolic features of HD. The mechanism of action of 3NP includes irreversible inhibition of succinate dehydrogenase (complex II) leading to mitochondrial dysfunction and selective striatal toxicity [71–76]. With this purpose, cortistatin-deficient mice (*Cort*^*−/−*^) and wild-type (*Cort*^*+/+*^) littermates received daily 3NP administration and were monitored to evaluate its susceptibility to mitochondrial-driven striatal injury. Our results revealed that *Cort*^*−/−*^ mice exposed to 3NP showed increased mortality, greater weight loss and impaired motor performance on the rotarod compared with 3NP-treated *Cort*^*+/+*^ controls (Fig. 2a and b).

**Fig. 3:**
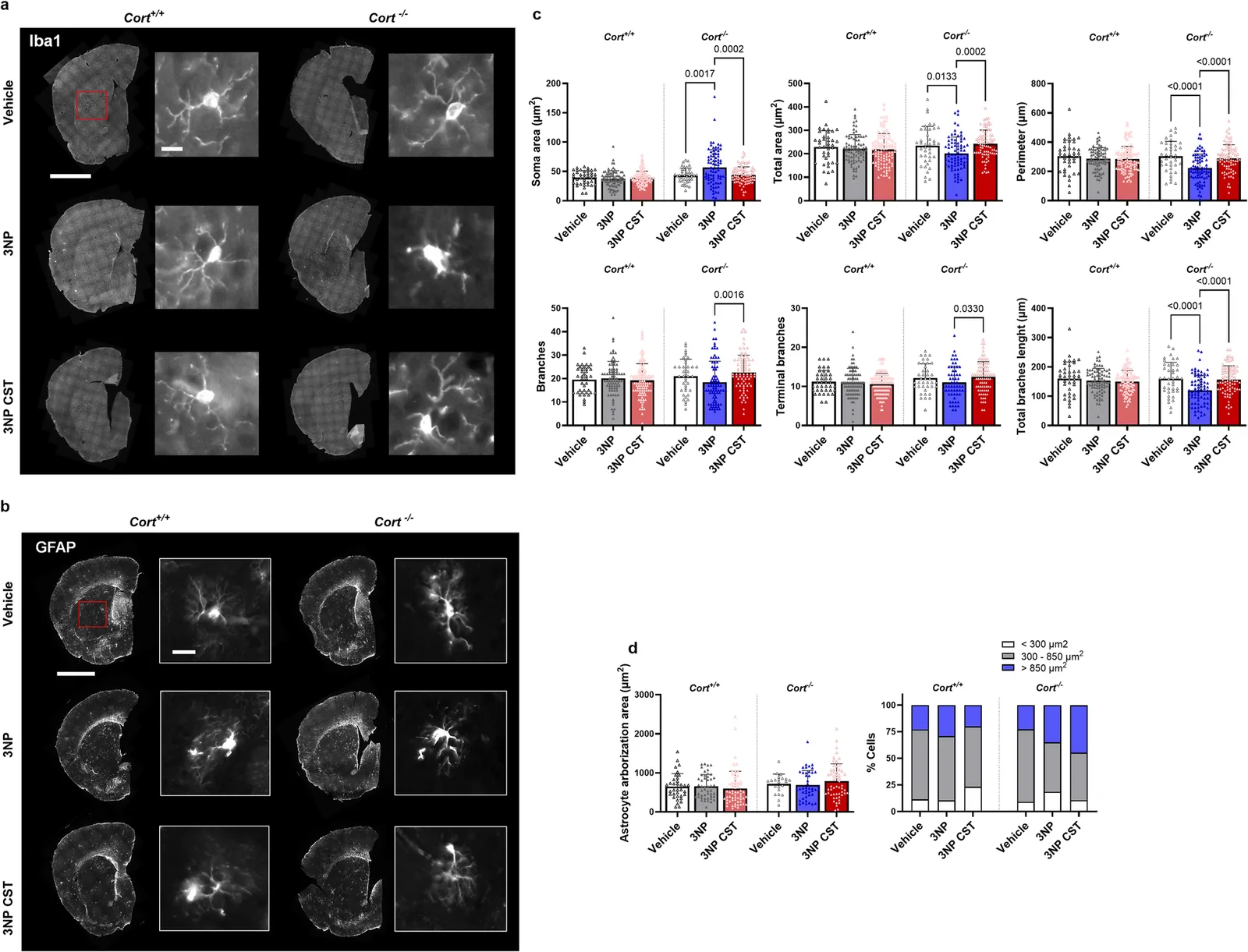
Cortistatin deficiency induces morphological changes in microglia and astrocytes in 3NP-treated mice. 3NP was intraperitoneally injected to *Cort*^*+/+*^ and *Cort*^*-/-*^ mice twice a day for 5 consecutive days (cumulated dose: 360 mg/kg; see methods), and glial responses were evaluated in the striatum (red square) after cortistatin (CST) treatment (0.3 mg/kg). **a**, **b** Representative coronal brain sections immunostained for Iba1 (white) to identify microglia (**a**) and for GFAP (white) (**b**) to identify astrocytes. Scale bar: 1500 µm (whole section) and 10 µm (higher magnification) for Iba1 and 20 µm (higher magnification) for GFAP. **c**, **d** Morphological characterization was performed using ImageJ Fiji free software. For Iba1-stained microglia (**c**), soma area was assessed manually, total area and perimeter were quantified using the “*Analyze Particles plugin*”, number and length of primary and terminal branches were determined by “*Skeleton Analysis plugin*”. For GFAP-stained astrocytes, morphological complexity was assessed by quantifying the astrocyte arborization area and distributing cells into three categories based on arborization area size: small (< 300 µm²), medium (300–850 µm²), and large (> 850 µm²) (**d**). *N* = 35-125 cells from 3-5 mice per experimental group. Data represent the mean ± SEM. One-way ANOVA followed with Fisher´s LSD post-hoc test. CST: cortistatin, GFAP: glial fibrillary acidic protein, Iba1: ionized calcium-binding adaptor molecule 1, 3NP: 3-nitropropionic acid

Although maximum survival in both genotypes ranged between 10 and 11 days, 50% mortality was reached earlier in *Cort*^*−/−*^ (day 7) than in control mice (day 9). Serum nitrite/nitrate concentrations, measured by Griess assay as indicators of systemic oxidative stress, were significantly elevated in 3NP-treated *Cort*^*−/−*^ mice but remained unchanged in *Cort*^*+/+*^ animals (Fig. 2c). Additionally, we evaluate white matter and axonal integrity in striatal brain sections that were immunostained for myelin basic protein (MBP) to label myelinated fibers. Western blot analysis of whole-hemisphere lysates did not reveal significant differences in MBP protein levels among all experimental groups (Fig. S3, Supplementary File 2). However, region-specific immunodetection of MBP in striatal sections showed that *Cort*^*−/−*^ mice treated with 3NP exhibited a marked reduction in MBP^+^ area and a decrease in the diameter of circular MBP^+^ profiles, which correspond to cross-sections of myelinated axon bundles (Fig. 2d-e). Regarding microglia, Iba1 immunostaining revealed that 3NP-treated *Cort*^−/−^ mice exhibited clear morphological signs of microglial reactivity, characterized by increased soma area alongside a reduction in total cell area, perimeter, number of branches, number of terminal branches, and total branch length (Fig. 3a, c). For astrocytes, morphological distribution analysis showed that 3NP increased the proportion of smaller GFAP^+^ cells in *Cort*^*−/−*^ mice, consistent with reactive remodeling (Fig. 3b, d). These alterations were not observed in 3NP-treated *Cort*^*+/+*^ mice, indicating selective vulnerability of the cortistatin-deficient striatum to demyelination, axonal injury and glial activation. In contrast, no significant differences were observed in Iba1^+^ or GFAP^+^ area in the striatum, nor in microglial fractal analysis parameters (Fig. S4a, b, Supplementary File 2). Interestingly, although exogenous cortistatin administration exerted only modest effects on weight loss and rotarod performance, it significantly restored serum nitrite/nitrate levels and normalized myelin and glial-related parameters in 3NP-treated Cort^−/−^ mice (Figs. 2b-e and 3). Collectively, these findings indicate that cortistatin deficiency heightens vulnerability to 3NP-induced striatal toxicity, and that cortistatin supplementation appears to confer partial protection against oxidative stress, myelin damage and glial reactivity.

### Cortistatin attenuates 3NP-induced inflammatory responses in mHTT-expressing striatal neurons

Based on the association between cortistatin deficiency and the manifestation of Huntington-like features, we decided to further investigate the contribution of cortistatin into two pathological processes central to HD: neuroinflammation and mitochondrial dysfunction. For this purpose, we employed immortalized striatal neurons derived from *Hdh*^*Q111/Q111*^ mice, as the striatum represents the region most severely affected in HD and these neurons exhibit marked cortistatin deficiency (Fig. 1c). We first characterize the basal inflammatory profile of *ST*^*Q111/Q111*^ neurons relative to wild-type striatal neurons (*ST*^*Q7/Q7*^), and subsequently we generate a more robust model to study HD pathology, by investigating the interaction between mHTT and cellular stress pathways induced by 3NP. Then, we use this tool to test cortistatin supplementation as a potential therapy.

**Fig. 4:**
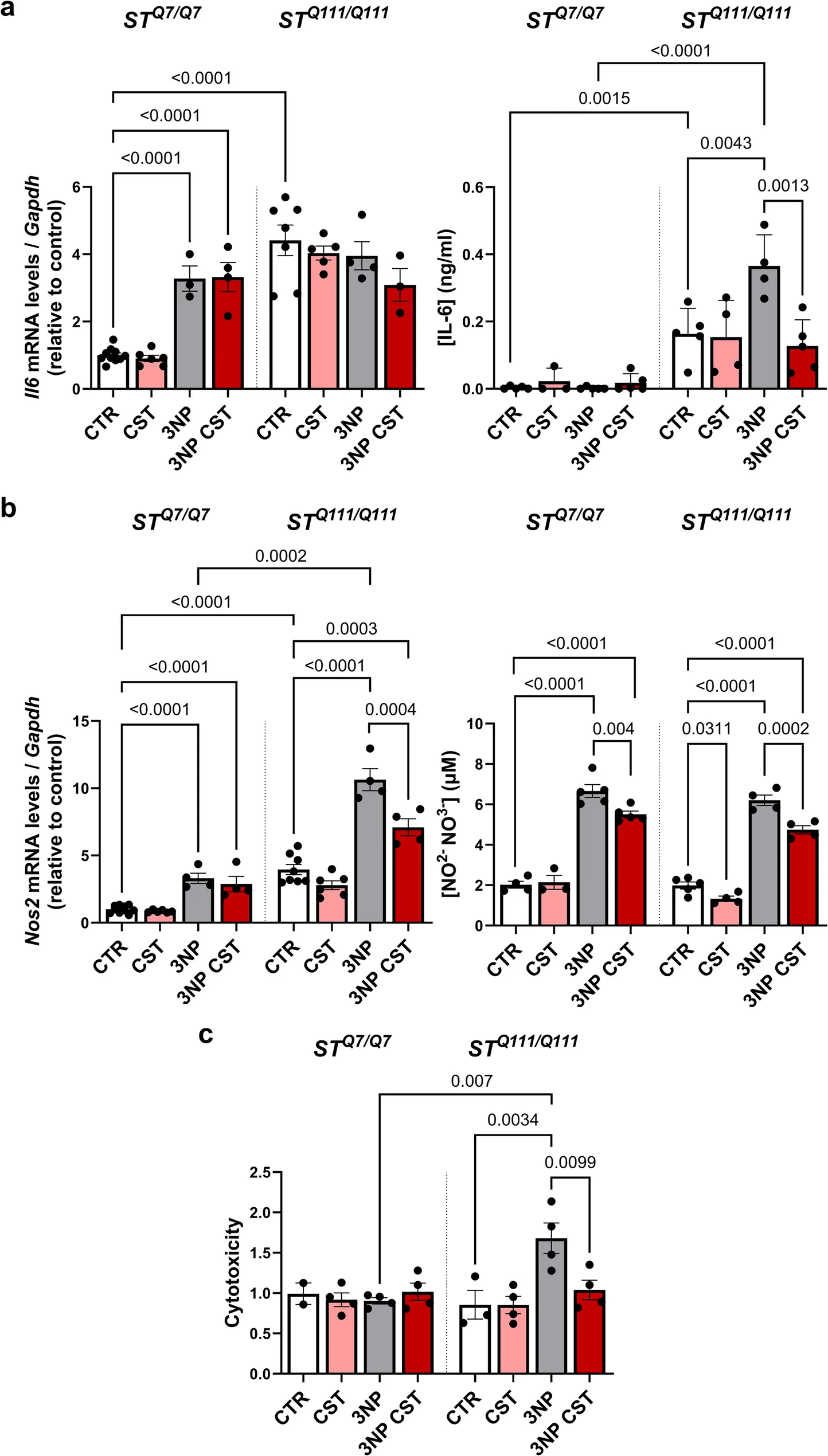
Cortistatin attenuates inflammatory and cytotoxic responses in mHTT-expressing striatal neurons. Wild-type (*ST*^*Q7/Q7*^) and mHTT (*ST*^*Q111/Q111*^) striatal neurons were treated with cortistatin (CST, 100 nM), and/or 3NP (2.5 mM) for 24 h. **a** Left, gene expression levels of *Il6* quantified by real-time qPCR and normalized to *Gapdh* expression (*N* = 3-10 cell cultures); right, IL-6 levels in culture supernatants were measured by ELISA (*N* = 3-5). **b** Left, gene expression levels of *Nos2* quantified by real-time qPCR and normalized to *Gapdh* expression (*N* = 4-10); Right, nitrite production in culture supernatants quantified by the Griess assay (*N* = 3-5). **c** Cell viability assessed after 24 h of treatment (*N* = 2-4). Data are presented as mean ± SEM, One-way ANOVA followed with Fisher´s LSD post-hoc test. Two-tailed unpaired t-test was used to compare *ST*^*Q7/Q7*^ CTR vs. *ST*^*Q111/Q111*^ CTR and *ST*^*Q7/Q7*^ 3NP vs. *ST*^*Q111/Q111*^ 3NP. CST: cortistatin, CTR: control, *Il6*/IL-6, interleukin-6, *Nos2*: inducible nitric oxide synthase, NO₂⁻ NO₃⁻, nitrites nitrates, 3NP: 3-nitropropionic acid

Baseline expression and production of IL-6, a proinflammatory cytokine detected in circulation of HD patients and experimental models, were significantly elevated in *ST*^*Q111/Q111*^ neurons compared with wild-type cells (Fig. 4a).

Exposure to 3NP induced a further increase in *Il6* expression in wild-type neurons but did not in *ST*^*Q111/Q111*^ neurons, potentially due to their already elevated baseline expression levels. Interestingly, although cortistatin treatment alone did not modify basal IL-6 expression or secretion, it significantly reduced 3NP-induced IL-6 production in *ST*^*Q111/Q111*^ neurons as well as initiated a clear trend toward reduced *Il6* expression (Fig. 4a).

Next, we analyzed *Nos2* expression (inducible nitric oxide synthase) as it contributes to inflammatory nitric oxide production. *Nos2* expression was significantly higher in *ST*^*Q111/Q111*^ neurons under basal conditions, although this was not accompanied by increased nitrite/nitrate levels (Fig. 4b). Administration of 3NP significantly increased both *Nos2* expression and nitrite/nitrate production in both cell genotypes. Cortistatin supplementation reduced nitrite/nitrate accumulation in *ST*^*Q111/Q111*^ basal neurons. Cortistatin also significantly reduced *Nos2* expression in *ST*^*Q111/Q111*^ neurons and lowered nitrite/nitrate accumulation in both wild-type and mutant neurons when combined with 3NP treatment (Fig. 4b). Consistent with these effects, cortistatin significantly decreased 3NP-induced cell death in *ST*^*Q111/Q111*^ neurons (Fig. 4c).

Additionally, we compared the anti-inflammatory actions of cortistatin with those of resveratrol and its derivative JC21, both previously reported to confer neuroprotection in models of 3NP-induced toxicity [50, 51]. While both resveratrol and JC21 could reduce IL-6 production in *ST*^*Q111/Q111*^ neurons, neither compound diminished 3NP-induced nitrite/nitrate levels, an effect uniquely achieved by cortistatin (Fig. S5a and b, Supplementary File 2).

Together, these results demonstrate that cortistatin attenuates proinflammatory activity and oxidative stress in striatal neurons expressing mutant huntingtin, which is consistent with potential neuroprotective role in the context of HD-associated inflammatory responses.

### Effects of cortistatin on mitochondrial bioenergetics

Next, we evaluated the influence of cortistatin on mitochondrial dysfunction, a well-established pathological feature in *ST*^*Q111/Q111*^ neurons [17, 62, 77–80]. First, we focused on mitochondrial bioenergetics, and we performed a Seahorse Cell Mito Stress Test following 3NP exposure and/or cortistatin supplementation in *ST*^*Q111/Q111*^ striatal neurons.

**Fig. 5:**
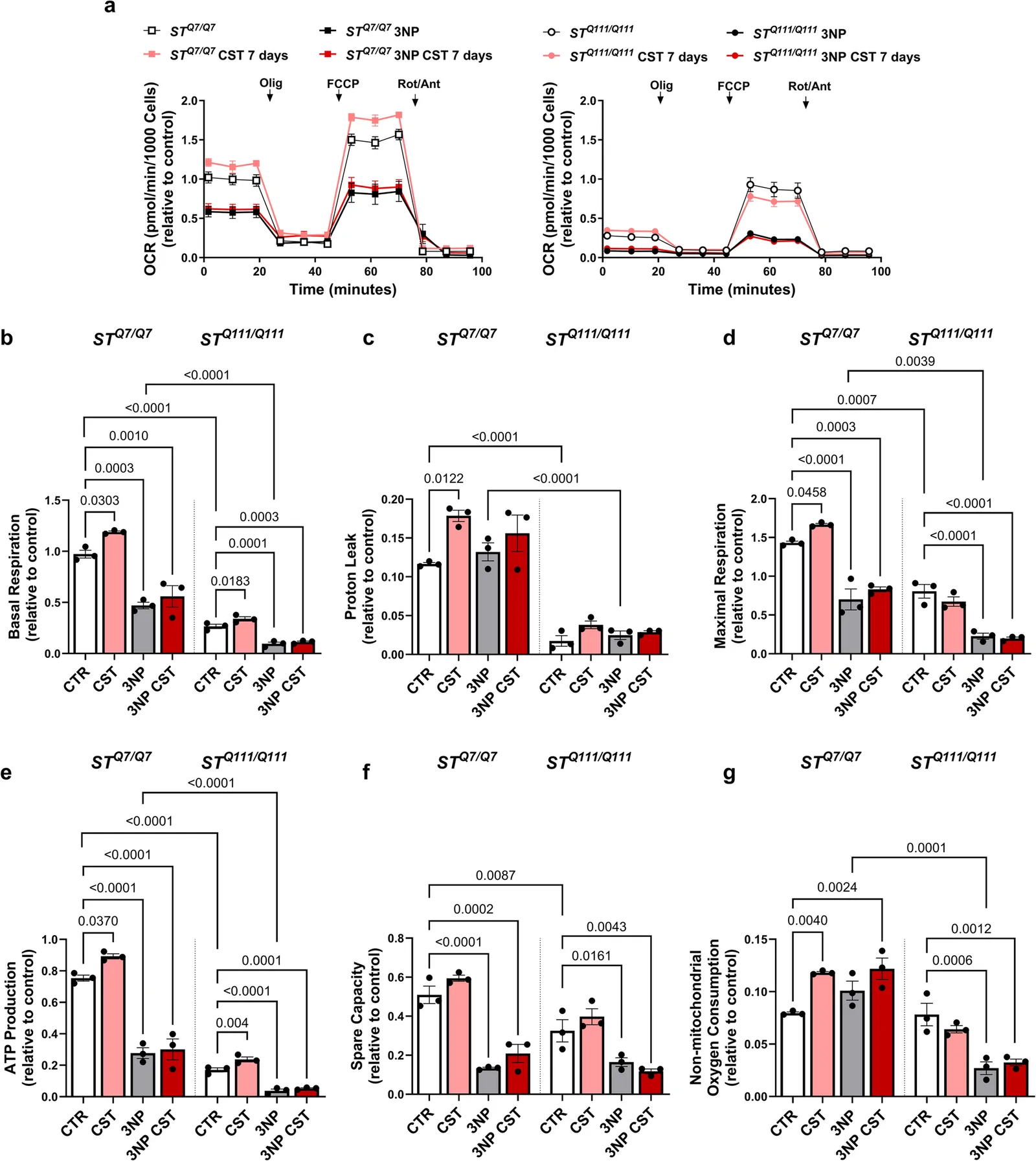
Long-term cortistatin treatment improves mitochondrial respiratory function in striatal neurons. Effects of cortistatin on mitochondrial bioenergetics in wild-type (*ST*^*Q7/Q7*^) and mutant huntingtin–expressing striatal neurons (*ST*^*Q111/Q111*^) treated with cortistatin (CST, 100 nM), and/or 3NP (2.5 mM) for 7 days. **a** Representative oxygen consumption rate (OCR) profiles obtained from Seahorse XF mitochondrial stress tests in *ST*^*Q7/Q7*^ (left) and *ST*^*Q111/Q111*^ (right) (*N* = 3 independent experiments). **b**-**g** Mitochondrial function was evaluated by analyzing the basal respiration (**b**), proton leak (**c**), maximal respiration (**d**), ATP production (**e**), spare respiratory capacity (**f**) and non-mitochondrial oxygen consumption (**g**) (*N* = 3). Data represent the mean ± SEM and are relative to the mean of the three measurements before oligomycin injection in *ST*^*Q7/Q7*^ CTR (normalized as 1). One-way ANOVA followed with Fisher´s LSD post-hoc test. Two-tailed unpaired t-test was used to compare *ST*^*Q7/Q7*^ CTR vs. *ST*^*Q111/Q111*^ CTR and *ST*^*Q7/Q7*^ 3NP vs. *ST*^*Q111/Q111*^ 3NP. Ant: antimycin A, CST: cortistatin, CTR: control, FCCP: carbonyl cyanide-p-trifluoromethoxyphenylhydrazone, Olig: oligomycin, Rot: rotenone, 3NP: 3-nitropropionic acid

Consistent with previous reports [78], *ST*^*Q111/Q111*^ neurons displayed marked respiratory impairment, including reduced basal respiration, proton leak, maximal respiration, ATP production, and spare respiratory capacity compared to wild-type neurons (Fig. S6a-g, Supplementary File 2). Nevertheless, individual complex II and IV activities remained unaltered (Fig. S6h, Supplementary File 2). In parallel, coenzyme Q_9_ (CoQ_9_) levels (mayor form of CoQ in mice) were significantly reduced in *ST*^*Q111/Q111*^, while CoQ_10_ levels remained unchanged (Fig. S6i, Supplementary File 2). On the other hand, mtDNA/nuDNA ratio was unaffected in neurons expressing mHTT (Fig. S6j, Supplementary File 2). Short-term cortistatin treatment (24 h) did not improve any of the mitochondrial bioenergetic parameters assessed in either genotype or did not mitigate 3NP-induced mitochondrial dysfunction, which further exacerbated the respiratory phenotype in mutant neurons (Fig. S6a-g, Supplementary File 2). Given the absence of short-term effects, we evaluated the consequences of prolonged cortistatin exposure. A 7-day treatment, cortistatin significantly enhanced basal respiration, proton leak, maximal respiration, and ATP production in wild-type neurons (Fig. 5a-e). In mutant neurons, extended cortistatin exposure increased basal respiration and ATP production (Fig. 5a, b, e). However, neither short- nor long-term cortistatin treatment conferred protection against 3NP-induced bioenergetic collapse, likely reflecting the severity of toxin-induced cellular damage (Fig. 5a-g, S6a-g, Supplementary File 2).

Since cortistatin’s effect on *ST*^*Q111/Q111*^ bioenergetics was limited and no increase in basal cell death was observed (Fig. 4c), we examined whether mHTT-expressing neurons might compensate for mitochondrial dysfunction through shifting their energy metabolism toward glycolysis. Glycolysis Stress Test analysis revealed that mutant neurons exhibited significantly higher basal glycolytic rates under glucose-rich conditions compared to wild-type neurons (Fig. S7a-c, Supplementary File 2). However, *ST*^*Q111/Q111*^ neurons showed reduced glycolytic capacity and glycolytic reserve (Fig. S7d, e, Supplementary File 2) indicating that while mutant neurons can meet basal energy requirements through enhanced glycolysis, they have limited capacity to upregulate glycolysis when faced with additional metabolic challenges. Cortistatin treatment did not modify glycolytic parameters in either cell genotype, supporting that cortistatin’s effects on cellular metabolism operate independently of glycolytic pathways.

### Timely protective effect of cortistatin against early oxidative stress in mHTT-expressing neurons

**Fig. 6:**
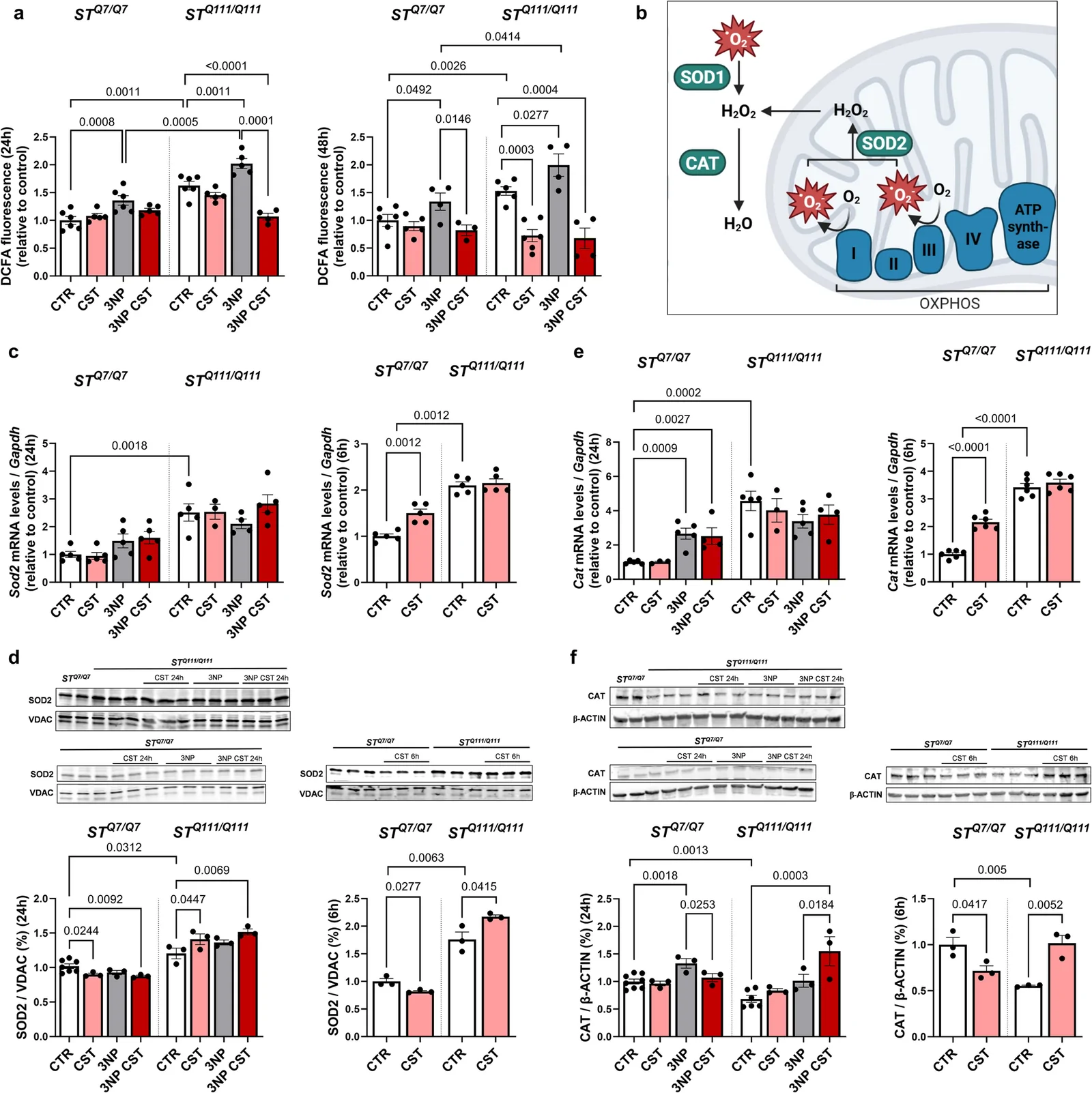
Cortistatin mitigates oxidative stress and enhances antioxidant defenses in mHTT-expressing neurons. Antioxidant effects of cortistatin in wild-type (*ST*^*Q7/Q7*^) and mHTT-expressing striatal neurons (*ST*^*Q111/Q111*^) treated with cortistatin (CST, 100 nM), and/or 3NP (2.5 mM). **a** Intracellular ROS levels measured by fluorimetry using the DCFH-DA fluorescent probe after 24 h (left) or 48 h (right) of treatment (*N* = 5). **b** Schematic representation of the enzymatic antioxidant defense system. SOD1, located primarily in the cytosol, and SOD2, located in the mitochondria, catalyze the conversion of superoxide anion (O₂·⁻) to hydrogen peroxide (H₂O₂), which is subsequently detoxified to water (H₂O) by CAT. Created with BioRender.com. Gene expression (**c**, **e**) and representative western blot images and protein bands quantification (**d**, **f**) for SOD2 and CAT. Levels of Sod2 (**c**) and Cat (**e**) quantified by real-time qPCR and normalized to Gapdh expression in *ST*^*Q7/Q7*^ and *ST*^*Q111/Q111*^ after 24 h of CST and/or 3NP treatment (left) or after 6 h of CST treatment alone (right). Protein levels of SOD2 (**d**) and CAT (**f**) quantified and normalized to VDAC or β-ACTIN in *ST*^*Q7/Q7*^ and *ST*^*Q111/Q111*^ after 24 h of CST and/or 3NP treatment (left) or after 6 h of CST treatment alone (right). (*N* = 3-6 cell cultures). Data represent the mean ± SEM. One-way ANOVA followed with Fisher´s LSD post-hoc test. Two-tailed unpaired t-test was used to compare *ST*^*Q7/Q7*^ CTR vs. *ST*^*Q111/Q111*^ CTR and *ST*^*Q7/Q7*^ 3NP vs. *ST*^*Q111/Q111*^ 3NP. *Cat*/CAT: catalase, CST: cortistatin, CTR: control, DCFH-DA: 2',7’-dichlorodihydrofluorescein diacetate, SOD1: superoxide dismutase 1, *Sod2*/SOD2: superoxide dismutase 2, VDAC: voltage-dependent anion channel 1, 3NP: 3-nitropropionic acid

Mitochondrial electron transport chain dysfunction represents a primary source of reactive oxygen species (ROS) in HD [62, 77, 78]. To determine whether cortistatin modulates the oxidative status in mHTT-expressing neurons, we quantified ROS following 3NP exposure and/or cortistatin supplementation. As expected, baseline ROS levels were significantly elevated in *ST*^*Q111/Q111*^ neurons, and 24 h exposure to 3NP further increased oxidative stress in both genotypes (Fig. 6a, right). Cortistatin treatment for 24 h did not reduce ROS under basal conditions; however, it significantly attenuated 3NP-induced ROS accumulation in mutant neurons. This effect became more pronounced after 48 h, at which point cortistatin normalized ROS levels in mutant neurons both with and without 3NP co-treatment, and in 3NP-treated wild-type neurons (Fig. 6a, left). We next compared the antioxidant effects of cortistatin with those of resveratrol and its derivative JC21, previously described as antioxidants [81–83]. After 24 h, cortistatin is the compound that most effectively reduced oxidative stress in both mutant and wild-type neurons exposed to 3NP (Fig. S5c, left, Supplementary File 2). By 48 h, all three compounds effectively decreased ROS levels in mHTT-expressing neurons under basal and 3NP conditions, affecting wild-type neurons only after 3NP exposure (Fig. S5c, right, Supplementary File 2). These findings suggest that cortistatin exerts earlier antioxidant activity than resveratrol or JC21. Because resveratrol has been reported to influence cortistatin expression [84], we evaluated whether the delayed antioxidant effects of resveratrol and JC21 could result from cortistatin upregulation. Gene expression analysis revealed no increase in cortistatin expression in response to either compound (Fig. S5d, Supplementary File 2), indicating that their antioxidant actions occur through cortistatin-independent mechanisms.

To further investigated the antioxidant pathways modulated by cortistatin, we evaluated the implication of SOD2 (superoxide dismutase 2), CAT (catalase) and SOD1 (superoxide dismutase 1), key mitochondrial and cellular antioxidant enzymes (Fig. 6b). SOD2 gene expression and protein levels were both elevated in *ST*^*Q111/Q111*^ neurons at baseline, consistent with a compensatory response to elevated oxidative stress (Fig. 6c, d). In contrast, although *Cat* mRNA was upregulated in mutant neurons, CAT protein levels were reduced (Fig. 6e, f). Cortistatin treatment for 24 h did not significantly modify the expression of these enzymes in either genotype (Fig. 6c, e, left). As there is typically an optimal window for transcriptional responses that may diminish over time as cellular homeostasis is restored, we performed gene expression analysis after 6 h of cortistatin treatment. Under these conditions, cortistatin induced a significant increase in *Sod2* and *Cat* expression in wild-type neurons but not in mutant cells (Fig. 6c, e, right). Interestingly, we detected an increase in SOD2 and CAT protein levels after cortistatin treatment for 6 h in *ST*^*Q111/Q111*^ neurons (Fig. 6d, f, right). The effect was also observed in mutant neurons following 24 h of cortistatin treatment and in combination with 3NP (Fig. 6d, f, left). Curiously, in wild-type neurons, cortistatin treatment for 6 h upregulated *Sod2* and *Cat* expression but decreased the protein levels of both antioxidant enzymes, suggesting cell-type- and context-dependent post-translational control (Fig. 6d, f). Finally, *Sod1* mRNA levels were elevated in *ST*^*Q111/Q111*^ and in both cell lines after 6 h of cortistatin treatment (Fig. S8a, Supplementary File 2). However, when SOD1 protein levels were assessed, no differences were observed between *ST*^*Q111/Q111*^ and control neurons, nor were any changes detected following cortistatin treatment or any other treatment condition (Fig. S8b, Supplementary File 2). The fact that cortistatin selectively modulates SOD2 and catalase at the protein level, but not SOD1, suggests that cortistatin-driven redox remodeling is compartment-specific, targeting the mitochondria-associated oxidative axis rather than the cytosolic one. In summary, our findings indicate that cortistatin can exert early protection against oxidative stress in mHTT-expressing neurons, operating through both rapid modulation of antioxidant enzyme expression and enhancement of cellular antioxidant capacity specially in the mitochondria.

### Intracellular signaling involved in the immunomodulatory and bioenergetic role of cortistatin

Given the well-established role of cAMP/PKA pathway in regulating both neuroinflammation and mitochondrial function in medium spiny neurons (MSNs) [85], we decided to investigate this intracellular mechanism as a potentially underlying pathway driving the effects of cortistatin. We found that basal intracellular cAMP levels were significantly lower in *ST*^*Q111/Q111*^ compared to wild-type neurons, and 3NP incubation did not significantly modify cAMP levels in either genotype (Fig. S9, Supplementary File 2). However, treatment with cortistatin increased cAMP levels in both striatal neurons under basal conditions, and in mutant neurons exposed to 3NP (Fig. S9, Supplementary File 2). Collectively, the pattern of cAMP reduction in *ST*^*Q111/Q111*^ neurons under conditions in which IL-6, nitrite, ROS, and cytotoxicity are elevated and ATP is diminished, together with the ability of cortistatin to simultaneously increase cAMP and reverse these pathological parameters, suggests that the cAMP/PKA pathway may constitute a relevant intracellular signaling axis involved in the immunomodulatory and bioenergetic effects of cortistatin in mHTT-expressing striatal neurons.

### Cortistatin restores mitochondrial network integrity under basal and stress conditions in striatal neurons

**Fig. 7:**
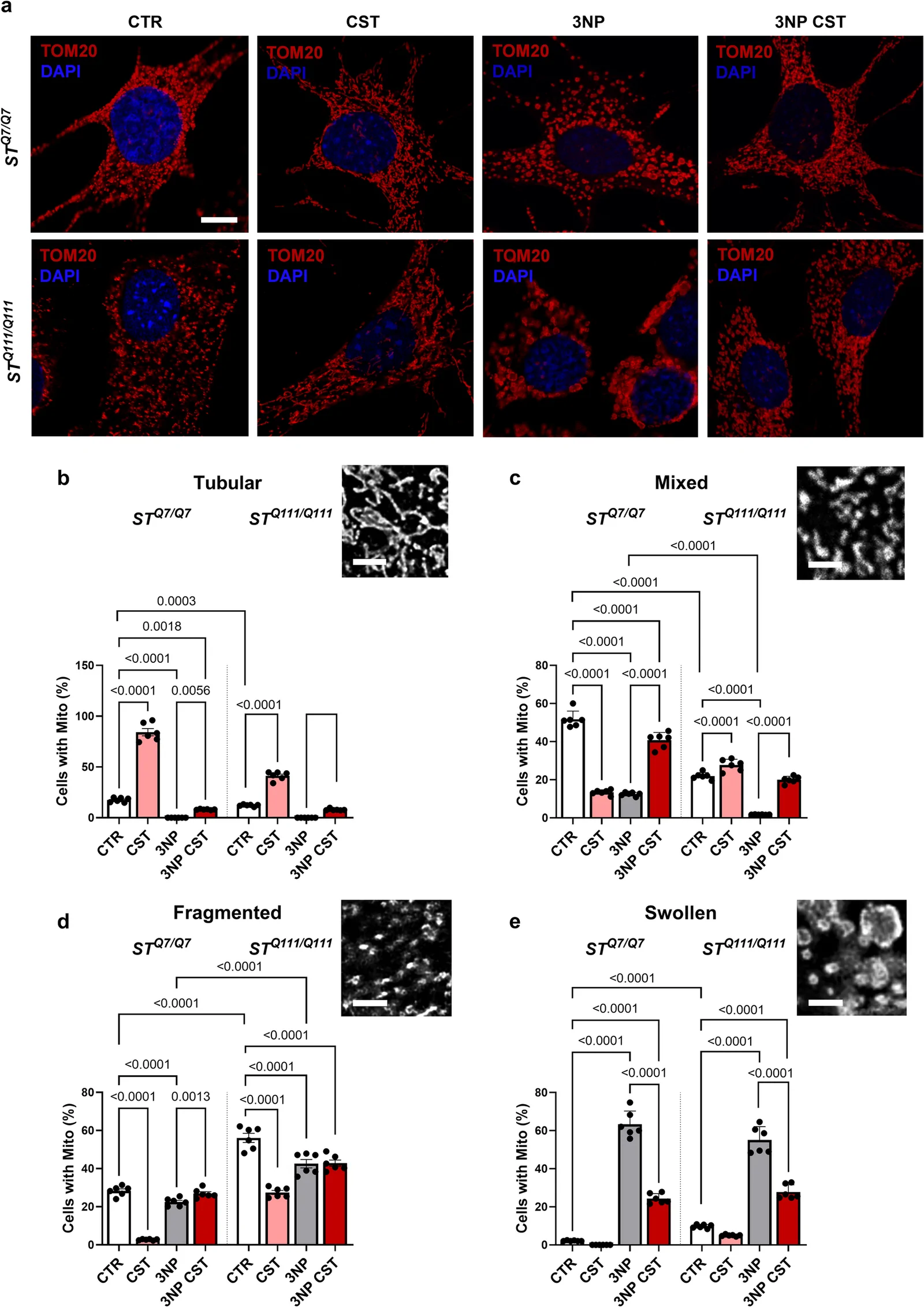
Cortistatin modulates mitochondrial morphology in striatal neurons. Regulation of mitochondrial morphology by cortistatin in wild-type (*ST*^*Q7/Q7*^) and mHTT-expressing striatal neurons (*ST*^*Q111/Q111*^) treated with cortistatin (CST, 100 nM), and/or 3NP (2.5 mM) for 24 h. **a** Representative confocal images illustrating predominant mitochondrial morphologies. Mitochondria were labeled with TOM20 (red), and classified by shape as tubular, mixed, fragmented and swollen. Nuclei were counterstained with DAPI (blue). Scale bar: 10 µm. **b**-**e** Quantification of the proportion of cells with each mitochondrial morphological category. Analyses were performed in 4-5 regions of interest (ROIs) per field, from 2 different fields per culture, across 3 independent experiments (*N* = 6 cultures/group). Scale bar: 3 µm. Data represent the mean ± SEM. One-way ANOVA followed with Fisher´s LSD post-hoc test. Two-tailed unpaired t-test was used to compare *ST*^*Q7/Q7*^ CTR vs. *ST*^*Q111/Q111*^ CTR and *ST*^*Q7/Q7*^ 3NP vs. *ST*^*Q111/Q111*^ 3NP. CST: cortistatin, CTR: control, DAPI: 4′,6-diamidino-2-phenylindole, TOM20: translocase of outer mitochondrial membrane 20, 3NP: 3-nitropropionic acid

Mitochondrial dynamics are highly sensitive to oxidative stress [86–90]. Given the elevated ROS levels observed in *ST*^*Q111/Q111*^ compared to wild-type, we investigated whether the antioxidant effects exerted by cortistatin were correlated with changes in mitochondrial morphology. With this purpose, mitochondrial architecture was assessed by TOM20 immunostaining (Fig. 7a).

Mitochondrial morphology was classified into four categories: tubular, fragmented, mixed, and swollen, according to established criteria [61, 62] (Fig. 7b-e). Under basal conditions, *ST*^*Q111/Q111*^ neurons predominantly exhibited fragmented mitochondria (56%), whereas wild-type neurons showed mainly mixed mitochondrial patterns (52%). Tubular mitochondria were infrequent in both genotypes (17% of *ST*^*Q7/Q7*^ and 12% of *ST*^*Q111/Q111*^ neurons), and swollen mitochondria were rare (2% and 9%, respectively) (Fig. 7). Tubular mitochondria facilitate efficient oxidative phosphorylation and cellular energy homeostasis while swollen mitochondria reflect compromised membrane integrity that ultimately leads to cellular energy deficit [91]. Cortistatin supplementation substantially shifted mitochondrial morphology toward the healthy tubular phenotype, significantly increasing tubular networks to 84% in wild-type and 40% in mutant neurons. In contrast, 3NP administration resulted in a dramatic accumulation of swollen mitochondria (65% in wild-type and 56% in mHTT neurons). Remarkably, cortistatin effectively counteracted the deleterious effects of 3NP, significantly reducing the proportion of swollen mitochondria while promoting the recovery of tubular and mixed morphological patterns in both genotypes (Fig. 7).

**Fig. 8:**
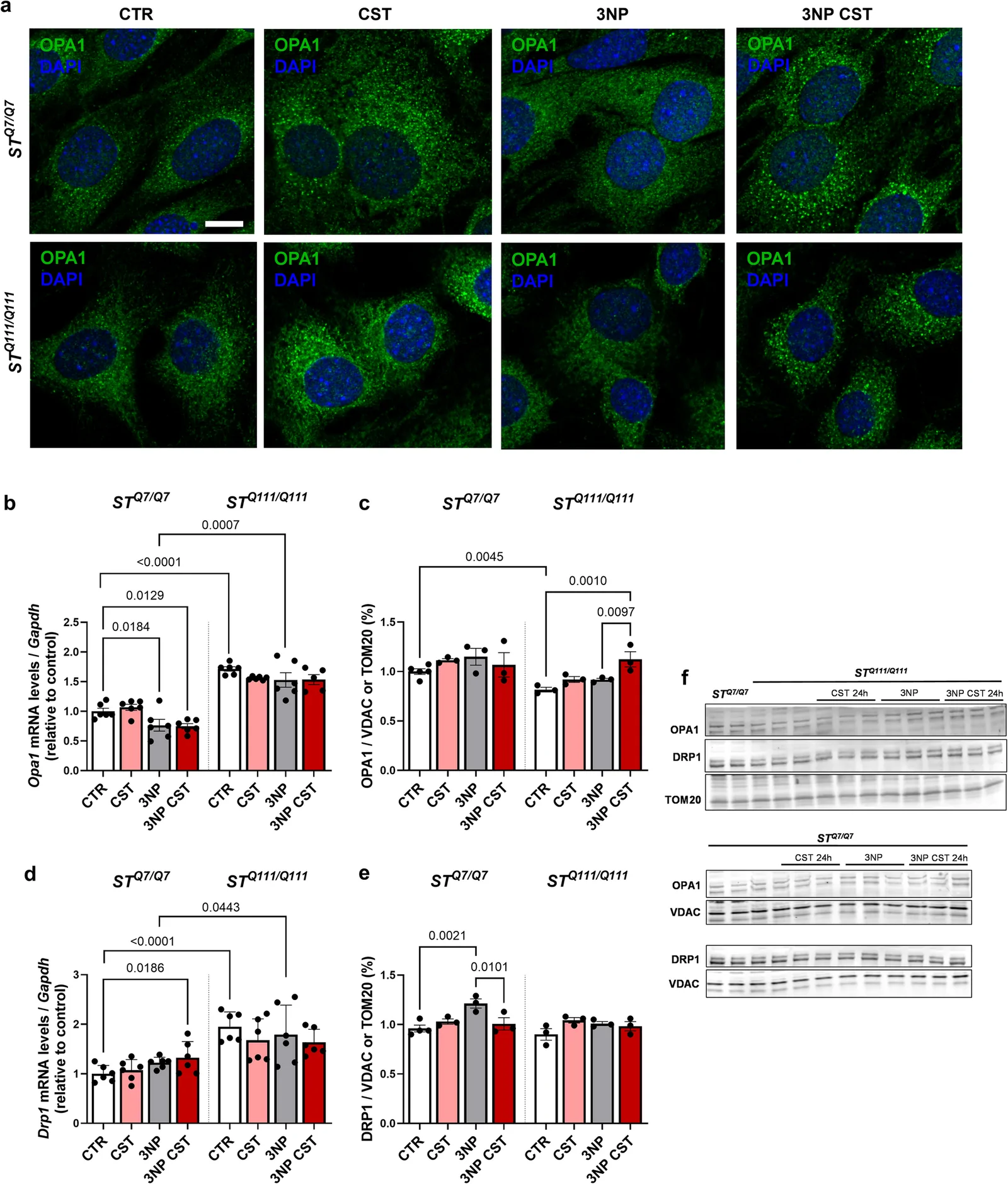
Cortistatin modulates mitochondrial dynamics in striatal neurons. Regulation of mitochondrial dynamics by cortistatin in wild-type (*ST*^*Q7/Q7*^) and mHTT-expressing striatal neurons (*ST*^*Q111/Q111*^) treated with cortistatin (CST, 100 nM), and/or 3NP (2.5 mM) for 24 h. **a** Representative confocal images of OPA1 immunostaining (green), with nuclei counterstained with DAPI (blue). Scale bar: 10 μm. Analyses were performed in 4 selected ROIs from 3 independent experiments (*N* = 3 cultures/group). **b**, **c** Gene expression of *Opa1*quantified by real-time qPCR and normalized to *Gapdh* expression (**b**) and protein bands of OPA1 quantified by western blot and normalized to VDAC or TOM20 (*N* = 3-6) (**c**). **d**, **e** Gene expression of *Drp1* quantified by real-time qPCR and normalized to *Gapdh* expression (**d**) and protein bands of DRP1 quantified by western blot and normalized to VDAC or TOM20 (*N* = 3-6) (**e**). **f** Representative western blot images of OPA1 and DRP1. Data represent the mean ± SEM. One-way ANOVA followed with Fisher´s LSD post-hoc test. Two-tailed unpaired t-test was used to compare *ST*^*Q7/Q7*^ CTR vs. *ST*^*Q111/Q111*^ CTR and *ST*^*Q7/Q7*^ 3NP vs. *ST*^*Q111/Q111*^ 3NP. CST: cortistatin, CTR: control, DAPI: 4′,6-diamidino-2-phenylindole, DRP1/*Drp1*: GTPase dynamin-related protein 1, OPA1/*Opa1*: optic atrophy 1, TOM20: translocase of the outer membrane 20, VDAC: voltage-dependent anion channel 1, 3NP: 3-nitropropionic acid

Subsequently, we investigated whether these morphological alterations were associated with changes in the mitochondrial fusion-fission machinery. For this purpose, we assessed OPA1 (optic atrophy type 1) localization by immunofluorescence. Our analysis revealed that cortistatin administration induced a pronounced shift from diffuse cytoplasmic distribution to a distinct punctate mitochondrial localization, particularly in cells co-treated with 3NP (Fig. 8a).

Gene expression analysis revealed a significant upregulation of *Opa1* and *Drp1* (GTPase dynamin-related protein-1) at baseline in *ST*^*Q111/Q111*^ neurons, but no significant changes were detected after cortistatin treatments (Fig. 8b, d). OPA1 protein levels were reduced in mutant neurons under basal conditions (Fig. 8c, f) but not DRP1 protein levels (Fig. 8e, f). Notably, cortistatin treatment in the presence of 3NP, significantly increased OPA1 protein expression selectively in mHTT-expressing neurons (Fig. 8c, f), potentially favouring mitochondrial fusion mechanisms and thereby contributing to the rebalancing of mitochondrial dynamics. In wild-type neurons, 3NP treatment alone induced a significant increase in DRP1 protein levels, consistent with the increased proportion of fragmented and swollen mitochondrial forms observed under this condition, an effect that was reversed upon cortistatin co-treatment (Fig. 8e, f). Collectively, these results indicate that the protective effects of cortistatin on mitochondrial morphology operate primarily through post-translational mechanisms, involving not only increased protein abundance but, most importantly, modulation of the subcellular localization and assembly of fusion machinery components, which are critical determinants of their functional efficacy.

**Fig. 9:**
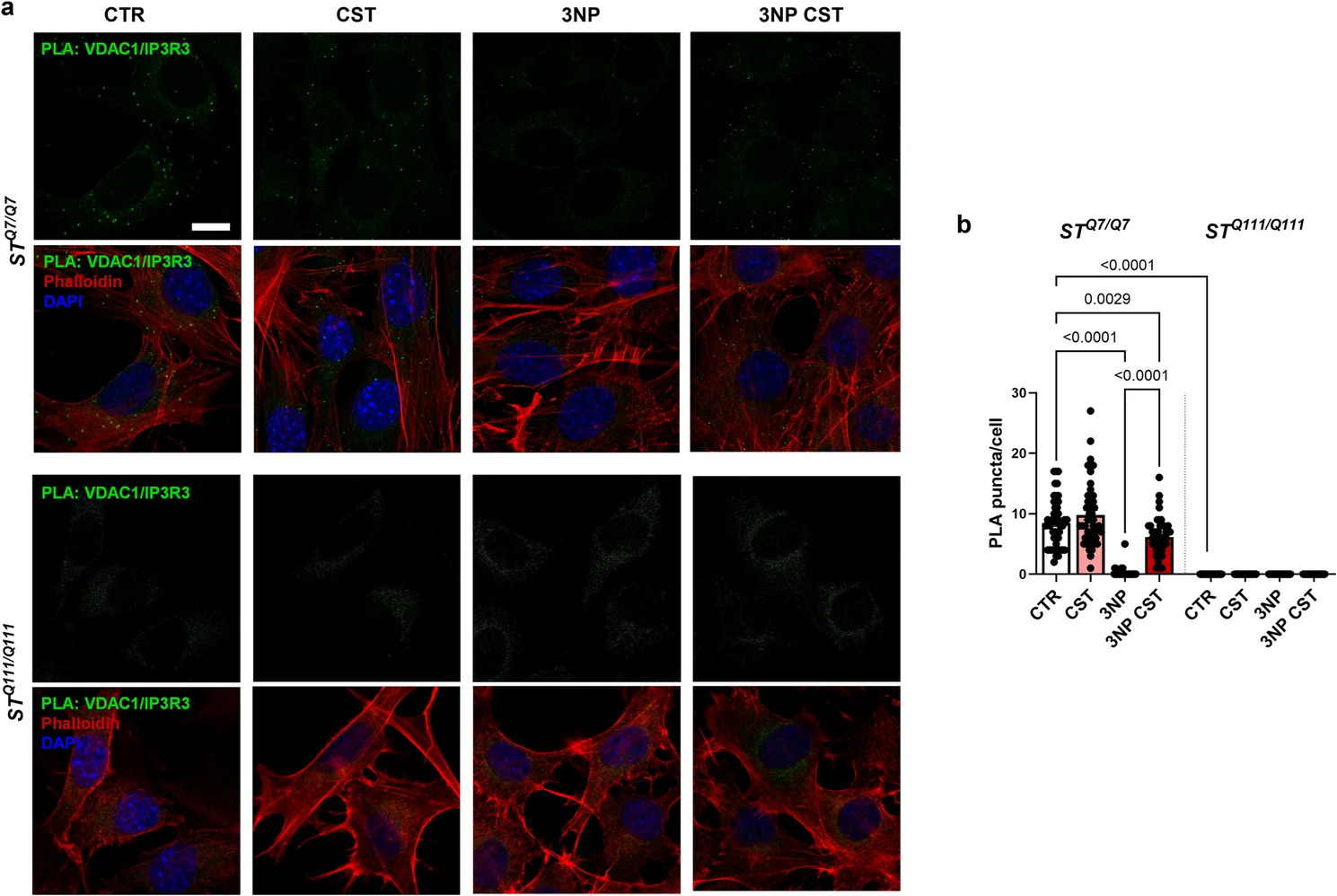
Cortistatin regulates mitochondria–endoplasmic reticulum interactions in striatal neurons. Regulation of mitochondria–endoplasmic reticulum (ER) contact sites by cortistatin in wild-type (*ST*^*Q7/Q7*^) and mHTT-expressing (*ST*^*Q111/Q111*^) striatal neurons treated with cortistatin (CST, 100 nM), and/or 3NP (2.5 mM) for 24 h. **a** Representative confocal images of proximity ligation assay (PLA) detecting mitochondria–ER interactions using VDAC and IP3R3 (green). Cell morphology was visualized with phalloidin staining (red), and nuclei were counterstained with DAPI (blue). Scale bar: 10 µm. **b** Quantification of PLA puncta per cell from 3 independent experiments (*N* = 47 cells/group). Data are presented as mean ± SEM. One-way ANOVA followed with Fisher´s LSD post-hoc test. Two-tailed unpaired t-test was used to compare *ST*^*Q7/Q7*^ CTR vs. *ST*^*Q111/Q111*^ CTR and *ST*^*Q7/Q7*^ 3NP vs. *ST*^*Q111/Q111*^ 3NP. CST: cortistatin, CTR: control, DAPI: 4′,6-diamidino-2-phenylindole, IP3R3: inositol 1,4,5-trisphosphate receptor type 3, VDAC1: voltage-dependent anion channel 1, 3NP: 3-nitropropionic acid

Alterations in mitochondrial morphology directly affect mitochondria-endoplasmic reticulum (ER) interactions, which play crucial roles in cellular homeostasis. To assess whether cortistatin could modulate mitochondria-ER contact sites, we performed proximity ligation assays (PLA) with VDAC1 as a marker for mitochondria and IP3R3 for ER. Our analysis revealed a notable absence of mitochondria-ER interactions in mutant huntingtin-expressing neurons under basal conditions, and neither cortistatin nor 3NP treatment modified this phenotype (Fig. 9a, b).

In contrast, wild-type neurons exhibited detectable mitochondria-ER contacts at baseline. Treatment with 3NP significantly reduced these interactions, consistent with metabolic stress-induced organellar dysfunction. Remarkably, co-administration of cortistatin with 3NP prevented this reduction, maintaining mitochondria-ER interaction levels comparable to untreated controls (Fig. 9a, b).

Together, these findings demonstrate that cortistatin improves mitochondrial morphology in mHTT-expression neurons under both basal and stress conditions, restores mitochondrial network integrity, and preserves mitochondrial-ER connectivity under metabolic stress, particularly in wild-type neurons, thereby potentially preserving essential inter-organellar communication pathways.

## Discussion

Huntington’s disease (HD) is a progressive neurodegenerative disorder in which mHTT triggers a complex cascade of pathogenic events extending beyond toxic protein aggregation [1, 2, 7, 8]. Neuroinflammation and mitochondrial dysfunction are now recognized as critical contributors to disease progression [11–16, 19–22, 24, 92, 93]. Current therapies remain symptomatic and fail to modify these core mechanisms [10], underscoring the need for disease-modifying approaches. In this context, cortistatin emerges as a potential candidate due to its combined anti-inflammatory and neuroprotective properties [35–39, 41–45]. Accumulating evidence showing that cortistatin deficiency promotes pro-inflammatory and oxidative phenotypes [29, 31, 41, 42, 44–46] prompted us to investigate its role as both a pathological determinant and a therapeutic agent in HD.

Our study identifies cortistatin as a previously unrecognized candidate regulator in HD (Graphical Abstract, Supplementary File 2). Transcriptomic reanalysis of human datasets revealed marked cortistatin downregulation in HD postmortem brains, particularly in the caudate nucleus, a region displaying early and severe neurodegeneration in HD [94, 95]. More modest alterations were detected in less vulnerable areas, suggesting that cortistatin deficiency may be associated with regional susceptibility. Importantly, dysregulation appeared specific to the cortistatin system, as related neuropeptides remaining unaffected, indicating targeted disruption rather than a general neurodegenerative response. Consistent reductions were detected in the striatum and serum of the *Hdh*^*Q7/Q111*^ mice, and in *ST*^*Q111/Q111*^striatal neurons, indicating that cortistatin deficiency is observed across human tissue, animal models, and cellular systems, and may represent a relevant feature of HD pathophysiology. In fact, the age-dependent reduction observed in *Hdh*^*Q7/Q111*^ mice, with significant effects emerging at 10 but not at 8 months, is consistent with disease progression and supports the idea that cortistatin loss accompanies worsening neuropathology. The regional specificity of the effect, with prominent reductions in striatum and hippocampus but not cortex, also reinforces the idea that cortistatin dysregulation tracks with selective vulnerability in HD. Similar cortistatin alterations described in other inflammatory and degenerative conditions further support its involvement in shared pathogenic pathways [40–42, 44–46]. Beyond changes in ligand availability, we identified alterations in cortistatin receptor expression. Reduced *SSTR1* and *SSTR4* expression was observed in human HD striatum and *Hdh*^*Q7/Q111*^ mice, whereas mHTT-expressing neurons displayed receptor upregulation. These divergent patterns likely reflect the influence of complex inflammatory and glial factors present in vivo but absent in in vitro conditions.

To determine functional consequences of cortistatin deficiency in HD pathogenesis, we used the 3-nitropropionic acid (3NP) model, which recapitulates key HD-like features, including selective striatal damage, motor impairment, and mitochondrial dysfunction [71–76, 96, 97]. Although neurotoxin models (such as 3NP) do not fully reproduce the genetic disease complexity, they remain valuable mechanistic tools for interrogating specific pathogenic pathways as 3NP model reproduces key pathological features of HD disease, including selective striatal neurodegeneration, motor dysfunction, neuroinflammatory changes, and bioenergetic impairment [98, 99]. Here, *Cort*^*−/−*^ mice exposed to 3NP exhibited exacerbated striatal pathology, behavioral impairment, increased nitrate/nitrite production, compromised myelin integrity and glial activation, indicating heightened inflammatory and structural damage and recapitulating neuroinflammatory signatures reported in genetic HD mouse models expressing mHTT, such as the R6/2 and Q175 as well as in postmortem human brain tissue from patients [22, 100, 101]. The lack of effect on MBP protein levels in mice’s brain is explained by the fact that demyelination is anatomically restricted to the striatum, a region that represents a minor fraction of the total brain hemisphere. Consequently, the striatum MBP signal is diluted by contributions from unaffected regions such as the cortex and corpus callosum, reducing the sensitivity of whole-hemisphere lysates to detect focal changes. In this context, our immunofluorescence analysis represents a more suitable approach, as it enables spatially resolved analysis within a defined region of interest, capturing not only signal intensity but also structural parameters of myelin integrity. The lack of changes regarding Iba1^+^ or GFAP^+^ cell density, despite clear morphological remodeling, likely reflects an early/mild reactive state. Indeed, the discrepancy between skeleton analysis (detecting branch retraction) and fractal dimension (preserved overall complexity) further supports localized rather than global architectural reorganization, consistent with subtle pathological remodeling rather than full activation. Complementary in vitro experiments demonstrated that 3NP-induced greater cytotoxicity, IL-6 production, and ROS generation in *ST*^*Q111/Q111*^ neurons, providing evidence that cortistatin dysregulation and mHTT expression synergistically amplify neurodegeneration. Importantly, cortistatin supplementation ameliorated these pathological alterations in both experimental systems.

Cortistatin has well-established immunomodulatory functions in central and peripheral disorders, regulating cytokine production, glial activity, and neurotrophic factors [36–38, 46, 102–106]. Given the central contribution of neuroinflammation to HD progression [19–21, 26], cortistatin-mediated modulation is particularly relevant. In our models, cortistatin reduced IL-6 production, attenuated nitrate/nitrite accumulation following 3NP administration, improved myelin and microglia-related parameters, and reduced neuronal cytotoxicity. Moreover, our findings highlight that the effects of cortistatin on astrocyte morphology are strongly context-dependent and reflect normalization toward homeostasis rather than opposite biological actions. In *Cort*^*+/+*^ mice, where endogenous cortistatin signaling is preserved, cortistatin appears to resolve reactive hypertrophy induced by 3NP, promoting a transition toward a more surveillant astrocytic phenotype. In contrast, in *Cort*^*−/−*^ mice, characterized by a heightened neuroinflammatory milieu and a mixed population of hypertrophic and atrophic astrocytes, cortistatin reduces the proportion of morphologically atrophic cells and restores arborization complexity. Thus, although cortistatin seem to induce opposite shifts in astrocyte size distribution between genotypes, the functional outcome is convergent, favouring recovery of a homeostatic astrocyte state. These results align with the current view of astrocyte reactivity as a heterogeneous and context-dependent spectrum [107] and underscore an important limitation of size-based morphological analyses, which do not fully resolve qualitative differences in astrocyte structure and function. Behavioral benefits were modest, likely reflecting the acute severity of 3NP toxicity and limited treatment window for cortistatin intervention [99, 108]. Whether cortistatin provides stronger benefits in chronic genetic HD models warrants future investigation.

Mitochondrial dysfunction is a central multifaceted pathogenic mechanism in HD [25], driven by direct mHTT interactions with mitochondrial proteins, altered mitochondrial transport and morphology, impaired ATP generation, and transcriptional dysregulation of mitochondrial regulators such as the transcriptional coactivator PGC-1α. This results in reduced mitochondrial biogenesis and impaired mitochondrial quality control mechanisms [109–111]. Here, we observed that *ST*^*Q111/Q111*^ neurons exhibited fragmented mitochondrial morphology, increased oxidative stress, and severely compromised respiratory capacity, consistent with previous observations of mHTT-driven disruption of mitochondrial dynamics and bioenergetics [62, 77–80, 99]. Fragmentation likely reflects mHTT-induced imbalance between fission and fusion processes, including DRP1 interaction and OPA1 reduction [62, 112–114]. Increased *Drp1* and *Opa1* transcripts in *ST*^*Q111/Q111*^ neurons may represent compensatory responses to mitochondrial stress but the lack of corresponding increase in protein levels suggests a state of transcriptional rigidity imposed by mutant huntingtin through its documented interference with mitochondrial gene networks [109, 115]. Despite preserved individual respiratory complex activity, mutant neurons showed reduced respiratory capacity and CoQ_9_ content, suggesting defective electron transport chain assembly and organization, and increased ROS production. The inefficient electron flow results in an increased tendency for electrons to react with molecular oxygen, forming ROS [116], a process also intensified by the accumulation of mHTT fragments in *ST*^*Q111/Q111*^ neurons [117, 118]. Under these conditions, antioxidant responses were insufficient to normalize ROS levels. Actually, we demonstrated a differential regulation of SOD2, catalase (CAT), and SOD1 at both genetic and protein expression, revealing compartment-specific antioxidant remodeling in mHTT-expressing neurons. Upregulation of SOD2 mRNA and protein in mutant neurons reflects a transcriptional adaptive response (typically mediated by the Nrf2/ARE and CREB pathways) to the elevated mitochondrial superoxide production driven by mHTT-induced bioenergetic failure [119]. On the other hand, the reduction of CAT protein despite elevated *Cat* mRNA is consistent with post-translational inactivation through substrate-mediated “suicide inhibition”, whereby the excess H_2_O_2_ generated downstream of heightened SOD2 activity leads to irreversible enzyme inactivation [120]. This effect is further compounded by impaired peroxisomal quality control secondary to mHTT-driven autophagy dysfunction [121, 122]. Finally, the dissociation between stress-responsive transcriptional induction and a stable protein pool observed for SOD1 in *ST*^*Q111/Q111*^ neurons can be explained by the intrinsic properties of this cytosolic metalloenzyme. Unlike SOD2, SOD1 protein levels are not primarily controlled at the transcriptional level but are instead determined by the availability of its copper cofactor and by the proteasomal degradation of any excess inactive protein. As a result, even when *Sod1* mRNA increases in response to oxidative stress, the total amount of SOD1 protein remains stable, since the system is already operating at its maximum metallation and assembly capacity [123, 124]. At the bioenergertic level, mutant neurons shifted toward glycolysis to maintain ATP production. Similar glycolytic enhancement has been reported in another neurodegenerative disorder, Alzheimer’s disease, where a Warburg-like metabolic transformation has been observed in fibroblasts and induced neurons derived from patient fibroblasts [125–127]. However, *ST*^*Q111/Q111*^ neurons demonstrated reduced glycolytic capacity and glycolytic reserve, indicating limited metabolic flexibility, which may increase vulnerability to additional energetic stress and contribute to the progressive neurodegeneration in HD.

Importantly, we demonstrated that the cortistatin deficiency detected in *ST*^*Q111/Q111*^ neurons was directly related to most of the mitochondrial defects described, as cortistatin supplementation reversed several mitochondrial abnormalities. Treatment improved mitochondrial morphology, increasing elongated tubular mitochondria under basal and 3NP conditions, in wild-type and mutant neurons, indicating that cortistatin promotes mitochondrial network integrity. These effects occurred independently of transcriptional regulation of *Opa1* or *Drp1*, suggesting post-transcriptional modulation. Indeed, cortistatin treatment increased the protein levels and mitochondrial localization of OPA1 and reduced its diffuse cytosolic pattern. The release of OPA1 into the cytosol has been implicated in cristae re-modeling leading to cytochrome c release and induction of mitochondrial fission [128]. This redistribution after cortistatin treatment indicates that the neuropeptide promotes the stabilization of fusion processes and preservation of cristae architecture, thereby limiting excessive fission. Even so, cortistatin treatment did not restore mitochondria-ER contacts in *ST*^*Q111/Q111*^ neurons. To date, it has been shown that aberrant DRP1-mediated mitochondrial fragmentation in R6/1 primary striatal neurons forces mitochondria away from the ER, thereby disrupting ER-mitochondria association. Notably, inhibition of DRP1 activity by Mdivi-1 treatment restored these contacts, linking the fusion-fission imbalance directly to mitochondria-associated membranes (MAM) disruption in this model [18]. In this regard, our data indicate that cortistatin restores mitochondrial morphology in *ST*^*Q111/Q111*^ neurons through modulation of OPA1 rather than DRP1, which may at least partially explain the failure to restore mitochondria-ER contact sites. Moreover, MAM integrity depends not only on mitochondrial morphology but also on the expression of resident tethering proteins, including IP3R3 and Grp75, both of which are significantly reduced in the striatum of HD animal models [18]. Importantly, our data from *ST*^*Q7/Q7*^ neurons provide a key mechanistic insight: in wild-type cells, cortistatin effectively rescues mitochondria-ER interactions following 3NP-induced mitochondrial stress, demonstrating its capacity to modulate MAM integrity under acute conditions. The absence of this rescue in *ST*^*Q111/Q111*^ neurons strongly suggests that mutant huntingtin engages additional pathogenic mechanisms at the MAM interface that are refractory to cortistatin treatment.

Mitochondrial morphological improvements in *ST*^*Q111/Q111*^ neurons were coupled to oxidative stress modulation. Although early effects at 24 h were modest, 48 h of cortistatin treatment drastically decreased ROS accumulation in mutant neurons, consistent with previous studies demonstrating a conserved antioxidative function of cortistatin across several disease models such as glucocorticoid-associated osteonecrosis and osteolysis [41, 42], septic cardiomyopathy [43] intervertebral disc degeneration [44] and diabetic ulcers [45]. In fact, in central intervertebral disc cells from *Cort*^*−/−*^ mice, increased ROS levels were associated with severe mitochondrial morphological damage and dysregulation of fusion/fission proteins including OPA1, DRP1, and MNF1/2. In our study, gene expression analysis suggested that, in mutant neurons, cortistatin likely enhances antioxidant capacity through post-translational regulation. This is supported by the observed increases in protein levels of SOD2, a mitochondria-localized enzyme, and CAT, which, although peroxisomal, is functionally coupled to the hydrogen peroxide generated by SOD2 activity. In this case, cortistatin increase CAT protein levels in *ST*^*Q111/Q111*^ by reducing the oxidative burden sufficiently to rescue the existing protein pool from inactivation. However, no corresponding changes were detected in SOD1 protein levels, a predominantly cytosolic antioxidant enzyme. This dissociation, considered alongside the distinct subcellular localization of these enzymes, is consistent with the possibility that cortistatin-mediated antioxidant remodeling in the mHTT context is compartment-specific, engaging primarily the mitochondrial-peroxisomal oxidative axis. This interpretation is supported by recent evidence that cortistatin modulates mitochondrial function [43, 44, 129], and it is particularly relevant given the pronounced mitochondrial dysfunction and elevated ROS production characteristic of *ST*^*Q111/Q111*^ neurons. It should be considered that additional redox systems, including glutathione peroxidases (GPXs) and peroxiredoxins (PRDXs) [130], may also contribute to the overall antioxidant cortistatin-mediated response. Additionally, shared receptor signaling with ghrelin raises the possibility of UCP2-dependent mitochondrial uncoupling mechanisms contributing to neuroprotection [131–134]. Supporting this mechanism, transgenic mice overexpressing UCP2 in catecholaminergic neurons exhibited neuroprotection and marked reduction in oxidative stress following acute MPTP exposure [135]. Together, these findings provide evidence for a working model in which cortistatin coordinates mitochondrial dynamics, redox balance, and inflammatory cascades, thereby limiting mHTT-mediated cellular damage. It should be noted, however, that these experiments were performed in immortalized striatal neurons, which may not fully recapitulate the electrophysiological and metabolic properties of primary medium spiny neurons or the complexity of the in vivo tissue environment.

Surprisingly, cortistatin failed to restore bioenergetic deficits in *ST*^*Q111/Q111*^ neurons despite improving mitochondrial morphology and oxidative stress. At first glance, these findings appear to contradict the well-established relationship between mitochondrial morphology and respiratory efficiency. Nevertheless, it is consistent with similar observations in other mitochondrial disorders where severe energetic compromise can occur independently of morphological alterations. For instance, fibroblasts from patients with mutations in β-oxidation enzymes showed a clear reduction in mitochondrial bioenergetics, especially under glucose deprivation conditions. However, these fibroblasts do not exhibit changes in mitochondrial morphology when examined using confocal and electron microscopy [136]. Our results highlight the complexity of mitochondrial regulation [137] and suggests that energetic impairment in mutant neurons may involve transcriptional dysregulation or metabolic alterations not directly targeted by cortistatin. Importantly, several therapeutic interventions in mitochondrial disorders provide neuroprotection without fully restoring bioenergetic parameters, supporting the clinical relevance of cortistatin-mediated protective mechanism [138–140].

Finally, our findings indicate that cortistatin is linked to a cAMP-dependent signaling deficit in HD striatal neurons and that exogenous cortistatin can partially reverse this defect. Although cortistatin acts though multiple receptors, activating diverse intracellular cascades [29], converging evidence points to the cAMP/PKA axis as a principal mediator of its neuroprotective and immunomodulatory actions [141–143]. This is particularly relevant in this study, as cAMP/PKA/CREB signaling is a well-established dysregulated pathway in HD models, especially in striatal neurons [144, 145], and restoring cAMP levels has been proposed as a therapeutic strategy [146]. The ability of cortistatin to restore cAMP levels suggests that its protective effect may involve re-engagement of an impaired prosurvival signaling pathway in *ST*^*Q111/Q111*^ neurons and supports the view that cortistatin deficiency may contribute to signaling collapse in HD. Within this framework, the improvements in the mitochondrial, oxidative, and neuroinflammatory pathological features of HD observed upon cortistatin treatment may not represent independent actions, but rather downstream transcriptional consequences of the restoration of the cAMP/PKA/CREB axis, though additional studies will be required to fully elucidate the processes involved.

In summary, this study identifies cortistatin as a potential modulator of HD pathogenesis and a promising therapeutic tool. Cortistatin is consistently downregulated across HD patient tissues and experimental models, and its deficiency aggravates inflammatory and mitochondrial dysfunction while increasing neuronal vulnerability to inflammatory and metabolic insults. Conversely, cortistatin supplementation attenuates inflammatory signaling, stabilizes mitochondrial integrity, reduces oxidative stress, and enhances cell viability. By targeting two interconnected pathological axes (neuroinflammation and mitochondrial instability), cortistatin represents a promising mechanism-based therapeutic strategy that may overcome limitations of current symptomatic treatments in HD. Moreover, these findings suggest that cortistatin-based strategies could have broader relevance for neurodegenerative diseases characterized by inflammatory and mitochondrial dysfunction.

## Supplementary Information

Supplementary Material 1.

Supplementary Material 2.

## Data Availability

The datasets supporting the conclusions of this article are available in the Gene Expression Omnibus database (GEO) repository, GSE51799 and GSE242195 in https://www.ncbi.nlm.nih.gov/geo/query/acc.cgi?acc=GSE51799 and https://www.ncbi.nlm.nih.gov/geo/query/acc.cgi?acc=GSE242195.

## Abbreviations

cAMP: Cyclic adenosine monophosphate
Cat: Catalase
CoQ: Coenzyme Q
CST: Cortistatin
DRP1: GTPase dynamin-related protein-1
GHRL: Ghrelin
GHSR: Ghrelin receptor
HD: Huntington’s disease
HTT: Huntingtin protein
IL-1β: Interleukin-1β
IL-6: Interleukin-6
mHTT: Mutant huntingtin protein
OPA1: Optic atrophy type 1
ROS: Reactive oxygen species
SST: Somatostatin
SSTR: Somatostatin receptors
Sod1: Superoxide dismutase 1
Sod2: Superoxide dismutase 2
TNFα: Tumor necrosis factor-α
TOM20: Translocase of outer mitochondrial membrane 20
3NP: 3-nitropropionic acid
