# Supplementary material for: Cortistatin as a modulator of inflammatory and mitochondrial dysfunction in Huntington´s disease
Source: J Neuroinflammation. 2026 Jul 7;23:329. doi: 10.1186/s12974-026-03947-4 (PMC13625329; doi:10.1186/s12974-026-03947-4)
Supplement: Supplementary file 1 [file 12974_2026_3947_MOESM1_ESM.docx]

**SUPPLEMENTARY MATERIAL FILE 1**

**Cortistatin as a modulator of inflammatory and mitochondrial dysfunction in Huntington´s disease**

Pilar González-García^1^*, Ignacio Serrano-Martínez^1^, Eliana Barriocanal-Casado^2,3^, Alejandro Cuenca-Martagón^1^, Pablo Vargas-Rodríguez^1^, Norma Adán^1^, Irene Forte-Lago^1^, Juan Carlos Morales^1^, Patricia Gomez-Suaga^4^, Júlia Bonet-Freixinet^5,6^, Silvia Ginés^5,6,7^, Luis C. López^2,3^, and Elena González-Rey^1^*

^1^Institute of Parasitology and Biomedicine Lopez-Neyra (IPBLN-CSIC), Granada, Spain; ^2^Department of Physiology, Faculty of Medicine, University of Granada, Granada, Spain;

^3^Institute of Biotechnology, Biomedical Research Center, University of Granada, Granada, Spain; ^4^Department of Biochemistry, Molecular Biology, and Genetics, Faculty of Nursing and Occupational Therapy, University of Extremadura, Cáceres, Spain;

^5^Departament de Biomedicina, Facultat de Medicina i Ciències de la Salut, Institut de Neurociències, Universitat de Barcelona, Barcelona, Spain;

^6^Institut d’Investigacions Biomèdiques August Pi i Sunyer (IDIBAPS), Barcelona, Spain;

^7^Networked Biomedical Research Center in Neurodegenerative Diseases - Carlos III Health Institute (CIBER-CIBERNED-ISCIII), Madrid, Spain.

***Correspondence**: elena.g@csic.es; pgonzalez@ipb.csic.es Tel.: +34-958-181670

**Membrane 1**. Original western blot membrane represented in Fig. 8 of OPA1 and DRP1, with TOM20 as loading control in mHTT-expressing striatal neurons (*ST^Q111/Q111^*) treated with cortistatin (CST, 100 nM), and/or 3NP (2.5 mM) for 24 h.

**Membrane 2**. Original western blot membrane represented in Fig. 6 of SOD2 with VDAC as loading control in mHTT-expressing striatal neurons (*ST^Q111/Q111^*) treated with cortistatin (CST, 100 nM), and/or 3NP (2.5 mM) for 24 h.

**Membrane 3**. Original western blot membrane represented in Fig. 6 and S8 of CAT and SOD1, with β-ACTIN as loading control in mHTT-expressing striatal neurons (*ST^Q111/Q111^*) treated with cortistatin (CST, 100 nM), and/or 3NP (2.5 mM) for 24 h.

**Membrane 4**. Original western blot membrane represented in Fig. 6 and 8 of SOD2 and DRP1, with VDAC as loading control in wild-type (*ST^Q7/Q7^*) striatal neurons treated with cortistatin (CST, 100 nM), and/or 3NP (2.5 mM) for 24 h.

**Membrane 5**. Original western blot membrane represented in Fig. 6, 8 and S8 of OPA1 with VDAC as loading control, and CAT and SOD1, with β-ACTIN as loading control in wild-type (*ST^Q7/Q7^*) striatal neurons treated with cortistatin (CST, 100 nM), and/or 3NP (2.5 mM) for 24 h.

**Membrane 6**. Original western blot membrane represented in Fig. 6 of SOD2 with VDAC as loading control in wild-type (*ST^Q7/Q7^*) and mHTT-expressing striatal neurons (*ST^Q111/Q111^*) treated with cortistatin (CST, 100 nM) for 6 h.

**Membrane 7**. Original western blot membrane represented in Fig. 6 and S8 of CAT and SOD1, with β-ACTIN as loading control in wild-type (*ST^Q7/Q7^*) and mHTT-expressing striatal neurons (*ST^Q111/Q111^*) treated with cortistatin (CST, 100 nM) for 6 h.

**Membrane 8**. Original western blot membrane represented in Fig. S3 of MBP with β-ACTIN as loading control in *Cort^+/+^* and *Cort^-/-^* 3NP-treated mice. *Cort^+/+^* groups and *Cort^-/-^* vehicle N = 2; *Cort^-/-^* groups N = 3.

**Membrane 9**. Original western blot membrane mice represented in Fig. S3 of MBP with β-ACTIN as loading control in *Cort^+/+^* and *Cort^-/-^* 3NP-treated mice*. Cort^+/+^* and *Cort^-/-^* vehicle N = 3; *Cort^+/+^* and *Cort^-/-^* treated-groups N = 2.
