# Supplementary material for: Cortistatin as a modulator of inflammatory and mitochondrial dysfunction in Huntington´s disease
Source: J Neuroinflammation. 2026 Jul 7;23:329. doi: 10.1186/s12974-026-03947-4 (PMC13625329; doi:10.1186/s12974-026-03947-4)
Supplement: Supplementary file 2 [file 12974_2026_3947_MOESM2_ESM.docx]

**SUPPLEMENTARY MATERIAL FILE 2**

**Cortistatin as a modulator of inflammatory and mitochondrial dysfunction in Huntington´s disease**

Pilar González-García^1^*, Ignacio Serrano-Martínez^1^, Eliana Barriocanal-Casado^2,3^, Alejandro Cuenca-Martagón^1^, Pablo Vargas-Rodríguez^1^, Norma Adán^1^, Irene Forte-Lago^1^, Juan Carlos Morales^1^, Patricia Gomez-Suaga^4^, Júlia Bonet-Freixinet^5,6^, Silvia Ginés^5,6,7^, Luis C. López^2,3^, and Elena González-Rey^1^*

^1^Institute of Parasitology and Biomedicine Lopez-Neyra (IPBLN-CSIC), Granada, Spain; ^2^Department of Physiology, Faculty of Medicine, University of Granada, Granada, Spain;

^3^Institute of Biotechnology, Biomedical Research Center, University of Granada, Granada, Spain; ^4^Department of Biochemistry, Molecular Biology, and Genetics, Faculty of Nursing and Occupational Therapy, University of Extremadura, Cáceres, Spain;

^5^Departament de Biomedicina, Facultat de Medicina i Ciències de la Salut, Institut de Neurociències, Universitat de Barcelona, Barcelona, Spain;

^6^Institut d’Investigacions Biomèdiques August Pi i Sunyer (IDIBAPS), Barcelona, Spain;

^7^Networked Biomedical Research Center in Neurodegenerative Diseases - Carlos III Health Institute (CIBER-CIBERNED-ISCIII), Madrid, Spain.

***Correspondence**: elena.g@csic.es; pgonzalez@ipb.csic.es Tel.: +34-958-181670

**Figure S1. Region-specific alterations of cortistatin-related genes in HD brains.** Cortistatin-related gene expression was evaluated across multiple brain regions in HD patients and healthy controls based on normalized RPKM values analyzed from the GEO transcriptomic dataset GSE242195. Gene expression levels of *CORT, SSTR1, SSTR4*, and *SSTR5* in the caudate nucleus **(a)** (N = 5-6 patients); in the cingulate cortex **(b)** (N = 7-10 patients); and in the nucleus accumbens **(c)** (N = 4-6 patients). All data are presented as the mean ± SEM, two-tailed unpaired *t*-test*. CORT*: cortistatin, HD: Huntington’s disease, *SSTR1*: somatostatin receptor 1, *SSTR4*: somatostatin receptor 4, *SSTR5*: somatostatin receptor 5.

**Figure S2.** **Cortistatin deficiency in HD models compromises neuronal viability.** Cortistatin downregulation and related gene expression changes were analyzed in peripheral blood, human brain tissue, mouse and *in vitro* cellular models. **(a)** Gene expression of *CORT* and *GHRL* in peripheral blood from HD patients (N = 76) and healthy controls (N = 25), extracted from the GEO dataset GSE51799, and expressed as normalized RPKM. **(b)** Gene expression of *SST*, *GHRL*, *SSTR2, SSTR3* and *GHSR* in postmortem brain tissue from HD patients (N = 20) and healthy controls (N = 17), from the GSE242195 dataset (expressed as normalized RPKM). **(c)**Tissue levels of cortistatin (ng/ml) in striatum (N = 4-6), hippocampus (HP, N = 6-7), and cortex (N = 3-6) of *Hdh^Q7/Q7^* and *Hdh^Q7/Q111^* mice at 8 (left) and 10 months of age (right) measured using ELISA assay. **(d)** Levels of cortistatin (ng/ml) in culture supernatants and cell viability of wild-type (ST*^Q7/Q7^*) and mHTT-expressing striatal neurons (ST*^Q111/Q111^*) after 24 h treatment with cytotoxic agents: 2.5 μM H₂O₂, 10 mM 3NP, 5 mM GLU and 0.5 μg/ml LPS. N = 3-5 independent experiments (except ST*^Q7/Q7^* vehicle with N = 2). Data represent the mean ± SEM, two-tailed unpaired *t*-test*.* *CORT*: cortistatin, *GHRL*: ghrelin, *GHSR*: growth hormone secretagogue receptor, GLU: glutamate, HD: Huntington’s disease, H₂O₂: hydrogen peroxide, HP: hippocampus, LPS: lipopolysaccharide, SST: somatostatin, *SSTR2*: somatostatin receptor 2, *SSTR3*: somatostatin receptor 3, 3NP: 3-nitropropionic acid.

**Figure S3.** **Myelin basic protein expression is preserved under 3NP treatment or cortistatin deficiency in mice.** 3NP was intraperitoneally injected to *Cort^+/+^* and *Cort^-/-^* mice twice a day for 5 consecutive days (cumulated dose: 360 mg/kg; see methods), and MBP protein levels were evaluated in the brain homogenates after cortistatin (CST) treatment (0.3 mg/kg). **(a)** Representative western blot images of MBP and protein bands quantification. N = 4-5 mice. Data represent the mean ± SEM. One-way ANOVA followed with Fisher´s LSD post-hoc test. CST: cortistatin, MBP: myelin basic protein, 3NP: 3-nitropropionic acid.

**Figure S4. Cortistatin deficiency does not affect the percentage of glial marked area or their fractal parameters in 3NP-treated mice.** 3NP was intraperitoneally injected to *Cort^+/+^* and *Cort^-/-^* mice twice a day for 5 consecutive days (cumulated dose: 360 mg/kg; see methods), and glial responses were evaluated in the striatum after cortistatin (CST) treatment (0.3 mg/kg). **(a)** Quantification of Iba1-positive and GFAP-positive area in the striatum. N = 3-5 mice (except *Cort^-/-^* vehicle with N = 2). **(b)** Fractal analysis was applied to obtain parameters including *fractal dimension, circularity* and *span ratio**.* N = 21-54 cells from 3-5 mice per experimental group. Data represent the mean ± SEM. One-way ANOVA followed with Fisher´s LSD post-hoc test. CST: cortistatin, GFAP: glial fibrillary acidic protein, Iba1: ionized calcium-binding adaptor molecule 1, 3NP: 3-nitropropionic acid.

**Figure S5. Cortistatin exhibits anti-inflammatory and antioxidant capacity in striatal neurons**. Striatal neurons were treated with cortistatin (CST,100 nM), and other anti-inflammatory/antioxidant compounds such as resveratrol (RES,10 μM) or compound JC21 (10 μM), with or without 3NP (2.5 mM) for 24 or 48 h. Top panels, wild-type (*ST^Q7/Q7^*) neurons; bottom panels, mHTT-expressing (*ST^Q111/Q111^*) striatal neurons **(a)** IL-6 levels in culture supernatants of wild-type and mHTT-expressing striatal neurons measured by ELISA after 24 h (N = 3-5 cell cultures) **(b)** Nitrite production in culture supernatants of wild-type and mHTT-expressing striatal neurons quantified by the Griess assay after 24 h (N = 3-5). **(c)** Intracellular reactive oxygen species (ROS) levels measured by fluorimetry using the DCFH-DA probe in wild-type and mHTT-expressing striatal neurons after 24 h (left panels) or 48 h (right panels) of treatment (N = 6). **(d)** Gene expression levels of *Cort* quantified by real-time qPCR and normalized to *Gapdh* expression in wild-type and mHTT-expressing striatal neurons after 24 h (N = 2-4). Data represent the mean ± SEM. One-way ANOVA followed with Fisher´s LSD post-hoc test. Two-tailed unpaired *t*-test was used to compare *ST^Q7/Q7^* CTR vs. *ST^Q111/Q111^* CTR and *ST^Q7/Q7^* 3NP vs. *ST^Q111/Q111^* 3NP. CST: cortistatin, CTR: control, DCFA: 2',7’-dichlorodihydrofluorescein diacetate, IL-6, interleukin-6, JC21: JC21 compound, NO₂⁻ NO₃⁻, nitrites nitrates, RES: resveratrol, 3NP: 3-nitropropionic acid.

**Figure S6.** **Short-term cortistatin treatment does not restore mitochondrial bioenergetics in mHTT-expressing neurons**. Effects of acute exposure (24 h) on mitochondrial function in wild-type (*ST^Q7/Q7^*) and mHTT-expressing (*ST^Q111/Q111^*) striatal neurons treated with cortistatin (CST, 100 nM), and/or 3NP (2.5 mM). **(a)** Representative oxygen consumption rate (OCR) profiles obtained from Seahorse XF mitochondrial stress tests in *ST^Q7/Q7^* (left) and *ST^Q111/Q111^* (right) (N = 3 independent experiments). **(b-g)** Mitochondrial function was evaluated by analyzing the basal respiration **(b)**, proton leak **(c)**, maximal respiration **(d)**, ATP production **(e)**, spare respiratory capacity **(f)** and non-mitochondrial oxygen consumption **(g)** (N = 3). **(h-j)** Activities of mitochondrial complex II **(h,** left panel**)**, and complex IV **(h,** right panel**)**, and levels of CoQ_9_ **(i**, left panel**)** and CoQ_10_ **(i,** right panel**)** following 24 h of CST treatment (N = 3). **(j)** Mitochondrial DNA to nuclear DNA (mtDNA/nDNA) ratio in wild-type and mHTT-expressing striatal neurons after 24 h CST treatment (N = 4). Data are presented as mean ± SEM. Data shown in a-g are represented as relative to the mean of the three measurements before oligomycin injection in *ST^Q7/Q7^* CTR (normalized as 1). One-way ANOVA followed with Fisher´s LSD post-hoc test. Two-tailed unpaired *t*-test was used to compare *ST^Q7/Q7^* CTR vs. *ST^Q111/Q111^* CTR and *ST^Q7/Q7^* 3NP vs. *ST^Q111/Q111^* 3NP. Ant: antimycin A, CII: complex II, CIV: complex IV, CoQ_9_: coenzyme Q_9_, CoQ_10_: coenzyme Q_10_, CST: cortistatin, CTR: control, FCCP: carbonyl cyanide-p-trifluoromethoxyphenylhydrazone, Olig: oligomycin, Rot: rotenone, 3NP: 3-nitropropionic acid.

**Figure S7.** **Cortistatin increases intracellular cAMP levels in striatal neurons.** Intracellular cAMP levels in wild-type (*ST^Q7/Q7^*) and mHTT- expressing (*ST^Q111/Q111^*) striatal neurons treated with cortistatin (CST, 100 nM), and/or 3NP (2.5 mM) for 1 h, or forskolin (10 μM) for 15 min, in the presence of phosphodiesterase inhibitor 3-isobutyl-1-methylxanthine (1 μM) (N = 4-6). Data are presented as mean ± SEM. One-way ANOVA followed with Fisher´s LSD post-hoc test. Two-tailed unpaired *t*-test was used to compare *ST^Q7/Q7^* CTR vs. *ST^Q111/Q111^* CTR. CST: cortistatin, CTR: control, FSK: forskolin, 3NP: 3-nitropropionic acid

**Figure S8.** **mHTT-expressing neurons exhibit compensatory enhancement of glycolytic metabolism**. Glycolytic profiling of wild-type (*ST^Q7/Q7^*) and mHTT- expressing (*ST^Q111/Q111^*) striatal neurons treated with cortistatin (CST, 100 nM), and/or 3NP (2.5 mM) for 24 h. **(a)** Representative graph of extracellular acidification rate (ECAR) obtained from Seahorse XF glycolysis stress tests (N = 3). Quantification of non-glycolytic acidification **(b)**, glycolysis **(c)**, glycolytic capacity **(d)**, and glycolytic reserve **(e)** (N = 3). Data are presented as mean ± SEM and are expressed as relative to the mean of the three measurements before glucose injection in *ST^Q7/Q7^* CTR (normalized as 1). One-way ANOVA followed with Fisher´s LSD post-hoc test. Two-tailed unpaired *t*-test was used to compare *ST^Q7/Q7^* CTR vs. *ST^Q111/Q111^* CTR. CST: cortistatin, CTR: control, Glu: glucose, Olig: oligomycin, 2-DG: 2-deoxy-D-glucose, 3NP: 3-nitropropionic acid.

**Figure S9.** **SOD1 is transcriptionally but not translationally regulated by cortistatin in striatal neurons.** Effects of cortistatin over SOD1 in wild-type (*ST^Q7/Q7^*) and mHTT-expressing striatal neurons (*ST^Q111/Q111^*) treated with cortistatin (CST, 100 nM), and/or 3NP (2.5 mM). **(a, b)** Gene expression levels of *Sod1* quantified by real-time qPCR and normalized to *Gapdh* expression **(a)** and representative western blot images of SOD1 and protein bands quantification (**b)** quantified by western blot and normalized to β-ACTIN in *ST^Q7/Q7^* and *ST^Q111/Q111^* after 24 h of CST and/or 3NP treatment (left) or after 6 h of CST treatment alone (right). N = 3-6 cell cultures. Data represent the mean ± SEM. One-way ANOVA followed with Fisher´s LSD post-hoc test. Two-tailed unpaired *t*-test was used to compare *ST^Q7/Q7^* CTR vs. *ST^Q111/Q111^*CTR and *ST^Q7/Q7^* 3NP vs. *ST^Q111/Q111^* 3NP. CST: cortistatin, CTR: control, *Sod1/*SOD1: superoxide dismutase 1, 3NP: 3-nitropropionic acid.

| **Gene** | **Primer sequence (5’-3’)** |
| --- | --- |
| *Cat* (NM_009804.2) | Fw: GGC ATC AAA AAC TTG CCT GT  Rv: TGG GTC AAA AGC CAT CTG TT |
| *Cst* (NM_001420759.1) | Fw: GCC TTC TGA CTT TCC TTG CC  Rv: GAA AGC TCC CCG CTG ATT GA |
| *Drp1 (NM_* *152816.4)* | Fw: GCC ATG CTG TCA ATT TGC TA  Rv: CGC TGC TTC TTT TCT TCG TT |
| *Gapdh (*NM_001411843.1*)* | Fw: AAC TTT GGC ATT GTG GAA GG  Rv: ACA CAT TGG GGG TAG GAA CA |
| *Ghsr* (NM_177330.4) | Fw: TCA GGG ACC AGA ACC ACA AA  Rv: CCA GCA GAG GAT GAA AGC AA |
| *iNos (NM_001313922.1)* | Fw: CTG CTG GTG GTG ACA AGC ACA TTT  Rv: ATG TCA TGA GCA AAG GCG CAG AAC |
| *Opa1 (NM_* *001199177.2)* | Fw: GCT CAG AAG ACC TTG CCA GT  Rv: CCT AAC AAG AGA AGG GCC TCA |
| *Sod1 (NM_* *011434.2)* | Fw: ACT TCG AGC AGA AGG CAA GC  Rv: AAA TGA GGT CCT GCA CTG GT |
| *Sod2 (NM_* *013671.3)* | Fw: ACA CAT TAA CGC GCA GAT CA  Rv: ATA TGT CCC CCA CCA TTG AA |
| *Sstr1* (NM_009216.3) | Fw: GCA AGA CGA CGC CAC CGT GA  Rv: CCG GCC TCA AAG CGT GCT GA |
| *Sstr2* (NM_001042606.2) | Fw: ATT TGT CCT GCT TAC TGT CG  Rv: CAG CTG TAC CAT CAA CTG GC |
| *Sstr3* (NM_009218.3) | Fw: AAG GGT CCA CGC CCA CGA AC  Rv: GCT AGC GCC AGC GGA TGT GT |
| *Sstr4* (NM_009219.3) | Fw: CAG GGA CAG CGG GCA TGG TC  Rv: AAG CAC TGC GCG ACA CAG CA |
| *Sstr5* (NM_011425.3) | Fw: TGG GGT GCA GCC TTC ATC ACT TAC  Rv: TGG CCG CCC ACT CTT GTC TG |

**Table S1. Sequence of primers used for real-time PCR quantifications.** Cat: Catalase, Cst: Cortistatin, Drp1: Dynamin-related protein 1, Gapdh: Glyceraldehyde-3-phosphate dehydrogenase, Ghsr: Growth hormone secretagogue receptor, iNos: Inducible nitric oxide synthase, Opa1: Optic atrophy 1, Sod1: Superoxide dismutase 1, Sod2: Superoxide dismutase 2, Sstr1: Somatostatin receptor 1, Sstr2: Somatostatin receptor 2, Sstr3: Somatostatin receptor 3, Sstr4: Somatostatin receptor 4, Sstr5: Somatostatin receptor 5

| **Day** | **Groups** | ***p*-value** |
| --- | --- | --- |
| 1 | *Cort^+/+^* 3NP vs *Cort^-/-^* 3NP | 0.017* |
|  | *Cort^+/+^* 3NP vs *Cort^-/-^* 3NP CST | 0.314 |
|  | *Cort^-/-^* 3NP vs *Cort^-/-^* 3NP CST | 0.006^$$^ |
| 2 | *Cort^+/+^* 3NP vs *Cort^-/-^* 3NP | 0.009** |
|  | *Cort^+/+^* 3NP vs *Cort^-/-^* 3NP CST | 0.004^++^ |
|  | *Cort^-/-^* 3NP vs *Cort^-/-^* 3NP CST | 0.008^$$^ |
| 3 | *Cort^+/+^* 3NP vs *Cort^-/-^* 3NP | 0.0005*** |
|  | *Cort^+/+^* 3NP vs *Cort^-/-^* 3NP CST | 0.007^++^ |
|  | *Cort^-/-^* 3NP vs *Cort^-/-^* 3NP CST | 0.054 |
| 4 | *Cort^+/+^* 3NP vs *Cort^-/-^* 3NP | 0.002** |
|  | *Cort^+/+^* 3NP vs *Cort^-/-^* 3NP CST | 0.0009^+++^ |
|  | *Cort^-/-^* 3NP vs *Cort^-/-^* 3NP CST | 0.280 |
| 5 | *Cort^+/+^* 3NP vs *Cort^-/-^* 3NP | 0.002** |
|  | *Cort^+/+^* 3NP vs *Cort^-/-^* 3NP CST | <0.0001^++++^ |
|  | *Cort^-/-^* 3NP vs *Cort^-/-^* 3NP CST | 0.0133^$^ |
| 6 | *Cort^+/+^* 3NP vs *Cort^-/-^* 3NP | 0.002** |
|  | *Cort^+/+^* 3NP vs *Cort^-/-^* 3NP CST | 0.0003^+++^ |
|  | *Cort^-/-^* 3NP vs *Cort^-/-^* 3NP CST | 0.033^$^ |
| 7 | *Cort^+/+^* 3NP vs *Cort^-/-^* 3NP | <0.0001**** |
|  | *Cort^+/+^* 3NP vs *Cort^-/-^* 3NP CST | 0.001^+++^ |
|  | *Cort^-/-^* 3NP vs *Cort^-/-^* 3NP CST | 0.008^$$^ |

**Table S2. Student’s t-test corresponding to Figure 2b body weight analysis**. *P*-values obtained from two-tailed unpaired Student’s t-test of *Cort^+/+^* 3NP, *Cort^-/-^* 3NP, and *Cort^-/-^* 3NP CST mice body weight recorded daily across 7 consecutive days. The tests correspond to the data presented in Figure 2b. 3NP (20 mg/kg – 60 mg/kg) and cortistatin (CST) (0.3 mg/kg) were given intraperitoneally daily over 7 consecutive days in *Cort^+/+^* and *Cort^-/-^* mice. Body weight was recorded daily. *Cort^+/+^* 3NP (N = 4), *Cort^-/-^* 3NP (N = 5), *Cort*^-/-^ 3NP CST (N = 5). CST: cortistatin, 3NP: 3-nitropropionic acid.

| **Day** | **Groups** | ***p*-value** |
| --- | --- | --- |
| 1 | *Cort^+/+^* 3NP vs *Cort^-/-^* 3NP | 0.711 |
|  | *Cort^+/+^* 3NP vs *Cort^-/-^* 3NP CST | - |
|  | *Cort^-/-^* 3NP vs *Cort^-/-^* 3NP CST | 0.673 |
| 2 | *Cort^+/+^* 3NP vs *Cort^-/-^* 3NP | 0.058 |
|  | *Cort^+/+^* 3NP vs *Cort^-/-^* 3NP CST | 0.079 |
|  | *Cort^-/-^* 3NP vs *Cort^-/-^* 3NP CST | 0.070 |
| 3 | *Cort^+/+^* 3NP vs *Cort^-/-^* 3NP | - |
|  | *Cort^+/+^* 3NP vs *Cort^-/-^* 3NP CST | - |
|  | *Cort^-/-^* 3NP vs *Cort^-/-^* 3NP CST | - |
| 4 | *Cort^+/+^* 3NP vs *Cort^-/-^* 3NP | 0.024* |
|  | *Cort^+/+^* 3NP vs *Cort^-/-^* 3NP CST | 0.058 |
|  | *Cort^-/-^* 3NP vs *Cort^-/-^* 3NP CST | 0.160 |
| 5 | *Cort^+/+^* 3NP vs *Cort^-/-^* 3NP | 0.004** |
|  | *Cort^+/+^* 3NP vs *Cort^-/-^* 3NP CST | 0.060 |
|  | *Cort^-/-^* 3NP vs *Cort^-/-^* 3NP CST | 0.023^$^ |
| 6 | *Cort^+/+^* 3NP vs *Cort^-/-^* 3NP | 0.0004*** |
|  | *Cort^+/+^* 3NP vs *Cort^-/-^* 3NP CST | 0.033^+^ |
|  | *Cort^-/-^* 3NP vs *Cort^-/-^* 3NP CST | 0.255 |

**Table S3. Student’s t-test corresponding to Figure 2b rotarod analysis**. *P*-values obtained from two-tailed unpaired Student’s t-test of *Cort^+/+^* 3NP, *Cort^-/-^* 3NP, and *Cort^-/-^* 3NP CST mice rotarod test recorded daily across 6 consecutive days. The tests correspond to the data presented in Figure 2c. 3NP (20 mg/kg – 60 mg/kg) and cortistatin (CST) (0.3 mg/kg) were given intraperitoneally daily over 7 consecutive days in *Cort^+/+^* and *Cort^-/-^* mice. *Cort^+/+^* 3NP (N = 4), *Cort^-/-^* 3NP (N = 5), *Cort*^-/-^ 3NP CST (N = 5). CST: cortistatin, 3NP: 3-nitropropionic acid.

**Graphical abstract.**  Cortistatin expression is reduced in Huntington's disease patients and experimental models of this pathology. In the 3-nitropropionic acid mouse model, cortistatin deficiency aggravates the disease hallmarks, while cortistatin treatment ameliorates them. These effects are mediated, at least in part, through the regulation of neuroinflammation and mitochondrial dysfunction, two key pathological mechanisms underlying HD progression. DRP1: Dynamin-related protein 1, HD: Huntington’s disease, IL-6: Interleukin-6, Nos2: Nitric oxide synthase 2, OPA1: Optic atrophy protein 1, ROS: reactive oxygen species, 3NP: 3-nitropropionic acid. Created with BioRender.com.
